# AuxB interacts directly with GpsB and PknB to coordinate cell envelope processes that contribute to intrinsic antibiotic resistance in *Staphylococcus aureus*

**DOI:** 10.1128/mbio.01858-25

**Published:** 2025-08-25

**Authors:** Tyler A. Sisley, Youngseon Park, Ace George Santiago, Wanassa Beroual, Isabella A. Sobolewski, Wonsik Lee, Joao A. Paulo, Suzanne Walker

**Affiliations:** 1 Department of Microbiology, Blavatnik Institute, Harvard Medical School 630058 , Boston, Massachusetts, USA; 2 School of Pharmacy, Sungkyunkwan University 105934, Suwon, Republic of Korea; 3 Department of Cell Biology, Harvard Medical School 168384 , Boston, Massachusetts, USA; Massachusetts Institute of Technology, Cambridge, Massachusetts, USA

**Keywords:** serine/threonine kinases, cell envelope, cell wall, *Staphylococcus aureus*, antibiotic resistance, PknB

## Abstract

*Staphylococcus aureus*, a leading cause of serious infections, produces various factors important for intrinsic resistance to antibiotics. Understanding what intrinsic resistance factors do may enable strategies to potentiate existing antibiotics. The membrane protein AuxB is an intrinsic resistance factor that helps *S. aureus* withstand diverse compounds that target the cell envelope, but its cellular functions are unknown. We show here that AuxB is a four-pass transmembrane protein with an intracellular C-terminus that interacts directly with the cytosolic cell cycle regulator GpsB. We also show AuxB’s membrane domain forms a homodimer that exists in equilibrium with a heterodimer of AuxB and PknB, a eukaryotic-like serine/threonine kinase that has been implicated in cell envelope processes. Shifting the equilibrium to favor AuxB-bound PknB impairs growth on tunicamycin, a condition where PknB is essential, which suggests that AuxB binding antagonizes a PknB function. To link PknB’s domains to compound susceptibility phenotypes, we assessed the fitness of PknB variants under several conditions. We find that PknB’s extracellular and kinase domains are not functionally interdependent but instead play distinct roles in withstanding cell envelope stress. AuxB evidently antagonizes functions of PknB’s extracellular PASTA (penicillin-binding protein and Ser/Thr kinase-associated) domain, the presence of which is beneficial under tunicamycin treatment regardless of whether the kinase domain is active. On compounds where the PASTA domain is deleterious, increasing the amount of AuxB-bound PknB can also ameliorate sensitivity. Collectively, our data suggest that AuxB, as a homodimer and through its interactions with GpsB and PknB, modulates cell envelope processes during cell growth and division.

**IMPORTANCE:**

*Staphylococcus aureus* is a leading cause of fatal infections worldwide. It encodes diverse genes that contribute to the organism's high intrinsic resistance to antibiotics. Understanding the biological roles of these genes and how their features contribute to intrinsic resistance may enable better antibiotic therapies. Here, we investigate AuxB, an intrinsic resistance factor to compounds that target the cell envelope. We find that AuxB interacts directly with the cell cycle regulator GpsB and the eukaryotic-like serine/threonine kinase PknB, another intrinsic resistance factor that is proposed to sense and respond to cell wall status. Based on our findings, we propose that AuxB impacts cell physiology through three mechanisms: (i) by antagonizing PknB's penicillin-binding protein and Ser/Thr kinase-associated domain function; (ii) by coordinating the phosphorylation of cell division proteins; and (iii) by forming a homodimer that interacts with GpsB hexamers to enable the formation of extended GpsB interaction networks.

## INTRODUCTION

**Fig 1:**
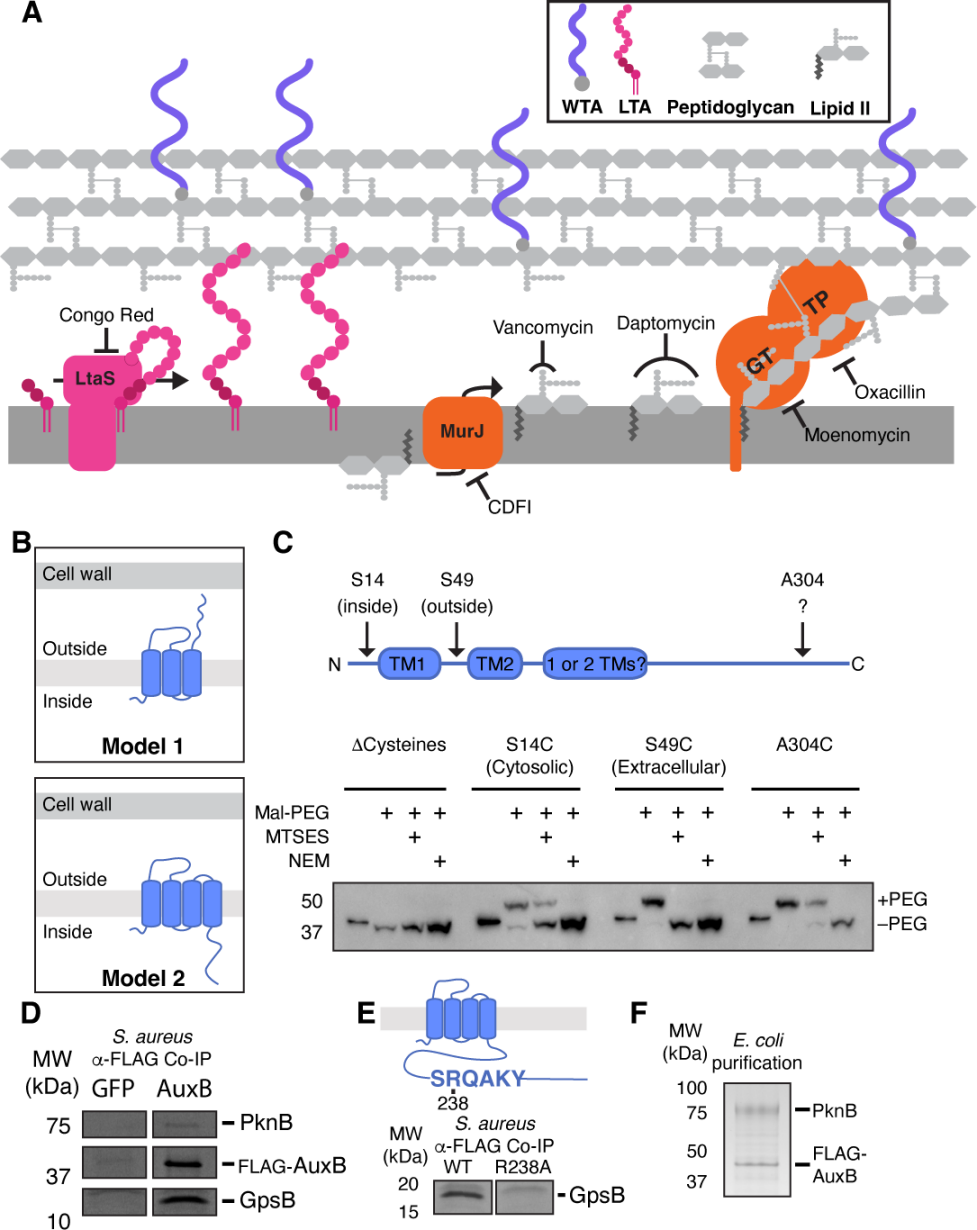
AuxB is a bacterial tetraspan that interacts with PknB and GpsB. (**A**) Diagram of the cell envelope in *S. aureus*. Relevant inhibitors and their targets are shown. GT, glycosyltransferase; TP, transpeptidase. (**B**) Model depicting the possible membrane topological organizations of AuxB predicted by TOPCONS (19). (**C**) Positions of amino acid replacements indicated along the model (top). Protoplasts were generated from cells expressing AuxB variants with single cysteine residues. Residues were blocked with MTSES (membrane impermeable) or NEM (membrane permeable) before labeling with maleimidyl-PEG. Migration differences were determined via a FLAG Western blot for FLAG-AuxB. (**D**) Coomassie-stained PAGE gel of co-immunoprecipitated FLAG-AuxB or FLAG-GFP. Labeled bands were identified via liquid chromatography-mass spectrometry (LC-MS) as PknB and GpsB. (**E**) AuxB contains an SRQAKY motif predicted to interact with GpsB (top). Coomassie-stained PAGE gel of GpsB from co-immunoprecipitation with AuxB^WT^ and AuxB^R238A^ (bottom). (**F**) FLAG-AuxB and 6xHis-PknB co-purify following expression in *Escherichia coli* and passage over αFLAG resin followed by size exclusion chromatography. Purified proteins were resolved by gel electrophoresis and Coomassie stain.

*Staphylococcus aureus* is a gram-positive pathogen that is responsible for a large fraction of deaths due to bacterial infections (1). It is therefore of utmost importance that we identify its vulnerabilities and understand how it withstands antibiotic stress. The first line of defense of *S. aureus* to hostile conditions is its multilayered cell envelope (2, 3), which contains numerous targets for antibacterial compounds (Fig. 1A). An essential layer of the cell envelope is the peptidoglycan cell wall, which protects cells from lysing under high turgor pressure (4). The cell wall is densely functionalized with anionic wall teichoic acids (WTAs), which contribute to intrinsic antibiotic resistance, and with various proteins important in physiology and virulence (5–8). Encapsulated by the cell wall is the cell membrane, the outer leaflet of which is rich in anionic lipoteichoic acids (LTAs) (9). Like wall teichoic acids, lipoteichoic acids play crucial roles in cell envelope integrity and intrinsic resistance to antibiotics (9–13). The cell membrane also harbors numerous enzymes involved in cell envelope biogenesis and a variety of proteins that sense and respond to changes in cell envelope status (14). Many non-essential *S. aureus* membrane proteins have been identified as intrinsic resistance factors (IRFs) because their presence contributes to the ability of *S. aureus* to withstand antibiotic stress (15, 16). Among these IRFs are the eukaryotic-like serine/threonine kinase PknB (Stk1) and the multipass membrane protein AuxB (SAOUHSC_01050) (15–18).

PknB is a single-pass membrane protein with an intracellular kinase domain and an extracellular domain comprising multiple PASTA (penicillin-binding protein and Ser/Thr kinase-associated) repeats (20). Orthologs of *S. aureus* PknB are widespread in *Bacillota* and in *Mycobacteriales* species (21–25). PknB is important for intrinsic resistance to β-lactam antibiotics and some other compounds that target the cell envelope (17, 18). From studies in *S. aureus* and other organisms, a model has emerged in which PknB senses cell envelope status and responds by phosphorylating proteins important in metabolism, cell envelope assembly, and cell division to modulate their activities (26–31). According to this model, PknB’s PASTA modules bind to, and dimerize around, cell wall fragments or precursors, which leads to kinase domain activation. Although there is strong evidence that PknB’s PASTA domain can bind lipid II and other cell wall muropeptides (32, 33), the evidence that kinase domain activation depends on the PASTA domain is inconclusive (26, 34, 35). Moreover, how different features of PknB impact susceptibility to cell envelope-targeting compounds has not been systematically examined.

AuxB has been far less studied than PknB. AuxB is a membrane protein that was identified from transposon sequencing (TnSeq) profiles as an IRF to β-lactams and other compounds that target the *S. aureus* cell envelope (15, 36, 37). One study has proposed a model to explain how AuxB confers intrinsic resistance (38). In this model, the positively charged C-terminal region of AuxB binds negatively charged lipoteichoic acids on the cell surface, which prevents lipoteichoic acid loss from the membrane. Because lipoteichoic acids contribute to resistance to some compounds that target the cell envelope (12, 39), this model is conceptually appealing; however, it has not been tested.

Here, we dissect how AuxB and PknB contribute to intrinsic antibiotic resistance. In testing the model that AuxB stabilizes lipoteichoic acids in the membrane, we discovered that AuxB’s C-terminal domain is intracellular and therefore cannot stabilize lipoteichoic acids through direct contact. Instead, we show that the intracellular C-terminal domain interacts with the cytosolic cell cycle regulator GpsB (40–45). We also show that the membrane domain of AuxB directly associates with PknB’s transmembrane (TM) helix and first PASTA repeat, and we find that this interaction antagonizes some PknB functions. By systematically probing the fitness of different PknB variants on cell envelope-targeting compounds, we find that PknB’s PASTA domain and kinase domain play distinct roles in the cell, and we show that PknB’s PASTA domain is not required for activity of the kinase domain. Taken together, the data are inconsistent with a model in which lipid II binding by the PASTA domain is required to activate PknB’s kinase domain and highlight an undiscovered function of the PASTA domain that is important under some stress conditions. We propose that AuxB forms dynamic complexes with GpsB and PknB to regulate their functions and, in doing so, coordinates cell envelope functions during growth and division.

## RESULTS

### AuxB is a four-transmembrane helix protein with an intracellular C-terminal domain

AuxB has a conserved transmembrane domain of unknown function (DUF4064) and a long C-terminal tail that is loosely conserved only in *Staphylococcaceae*. Topological prediction algorithms identified two possible models for AuxB—a three-TM helix model in which the C-terminal half of the protein is extracellular and a four-TM helix model in which this C-terminal region is intracellular (Fig. 1B). Based on the three-TM helix model and Δ*auxB*’s sensitivity to the lipoteichoic acid synthase inhibitor Congo Red, a previous study proposed that AuxB uses its extracellular C-terminal region to stabilize lipoteichoic acids in the membrane (38). Although depletion of LTAs from the membrane increases susceptibility to some compounds that target the cell envelope (5, 39), there were inconsistencies with this model. For example, loss of WTAs from the cell envelope is synthetically lethal with loss of LTAs (10, 46, 47), so the proposed model would predict a synthetically sick phenotype when Δ*auxB* cells are treated with a WTA inhibitor. However, ΔauxB cells are not susceptible to WTA inhibition (36).

As a starting point for understanding its functions, we decided to test AuxB’s topology using the substituted cysteine accessibility method (SCAM) (48). We pretreated cells with a membrane-impermeant, cysteine-reactive blocking agent, then lysed the cells and treated the lysates with a cysteine-reactive mass tag. Under these conditions, we observed a mass shift for an AuxB variant with a cysteine in its C-terminal region, showing that this region is intracellular (Fig. 1C). From this, we concluded that the predicted four-TM helix model is correct, consistent with the assignment of DUF4064 to the protein family database (Pfam) clan tetraspanin, which includes eukaryotic proteins that canonically have four-transmembrane helices (49–52).

### AuxB interacts with GpsB and PknB

We sought to identify protein-binding partners that might provide mechanistic clues about AuxB’s functions. To do so, we constructed a functional tagged allele of *auxB* (Fig. S1A) and immunoprecipitated it from *S. aureus*; when we analyzed eluates, we observed two bands on a Coomassie-stained gel that were not present in control samples (Fig. 1D and Fig. S1B, ~15 kD and ~75 kD). Mass spectrometry identified the high-molecular-weight binding partner as PknB and the low-molecular-weight binding partner as GpsB, a cell cycle regulator (40, 41) that is conserved in *Bacillota* (Tables S1 and S2). We searched for AuxB sequences similar to reported GpsB-binding motifs (53) and identified the sequence SRQAKY in the intracellular C-terminal region of the protein. Internal deletions that disrupted this sequence, or in which the motif’s Arg was replaced with Ala, no longer co-immunoprecipitated with GpsB (Fig. 1E and Fig. S1C). We concluded that AuxB uses its SRQAKY motif to bind GpsB. Because variants in which the SRQAKY motif was disrupted still formed a complex with PknB (Fig. S1D), we also concluded that AuxB interacts with PknB independently of GpsB. Consistent with this, we were able to co-purify the AuxB-PknB complex after heterologous co-expression of only these two proteins in *E. coli* (Fig. 1F).

We next tested whether GpsB binding was required for AuxB to maintain intrinsic resistance to antibiotics. The Δ*auxB* mutant was sensitive to Congo Red, daptomycin, vancomycin, and oxacillin, but cells expressing the Arg238Ala AuxB variant grew as well as wild type (WT) under all these conditions (Fig. S1A). We concluded that GpsB binding was not required for AuxB to function as an IRF under the tested conditions; we therefore shifted our focus to understanding AuxB’s interaction with PknB.

### AuxB forms a homodimer that competes with a PknB-AuxB heterodimer

**Fig 2:**
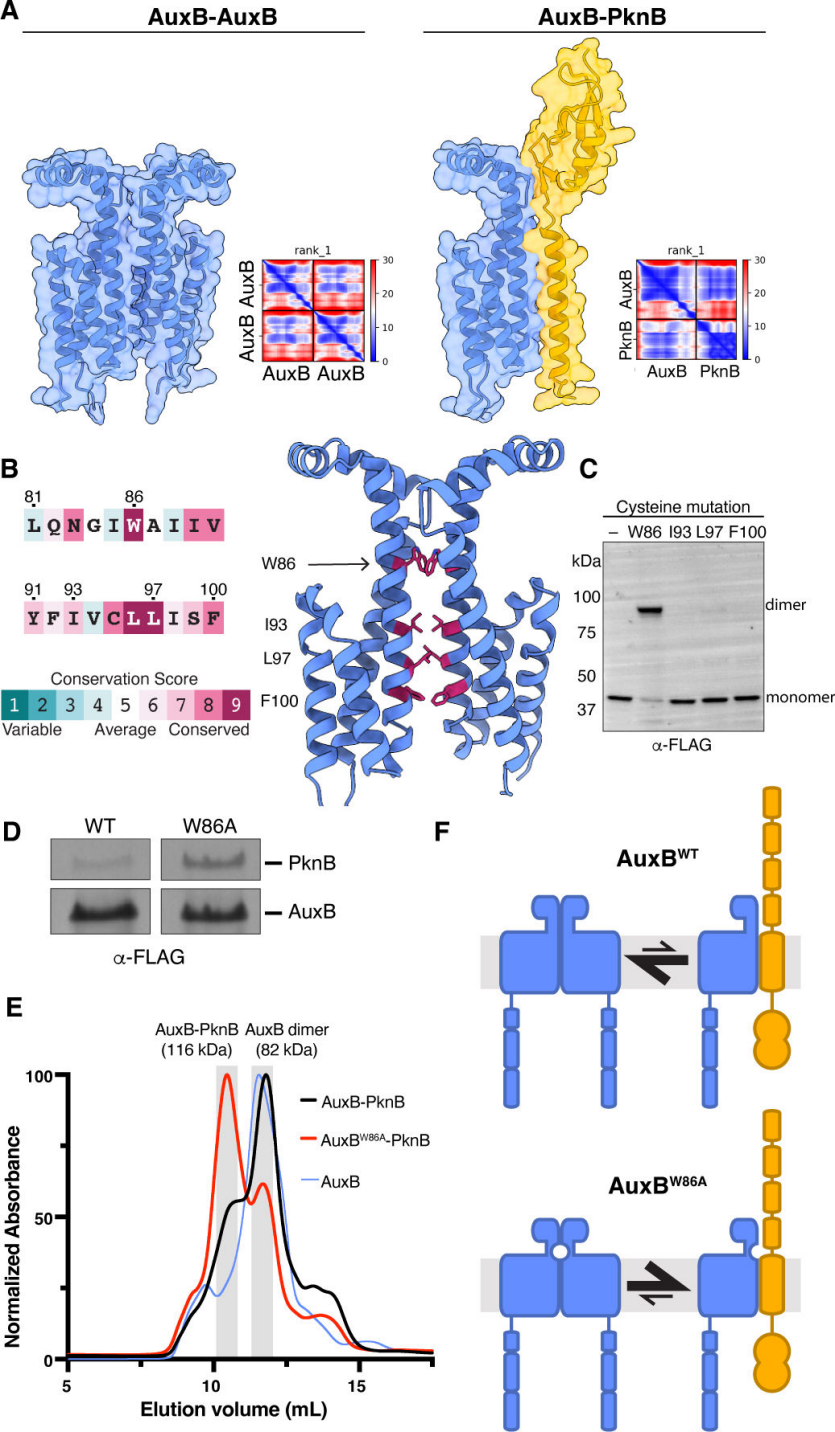
An AuxB homodimer occludes binding to PknB. (**A**) AlphaFold models and predicted alignment error plots of the AuxB-AuxB (left) and AuxB-PknB (right) complexes. (**B**) ConSurf (57–60) conservation scores for residues in AuxB TM helix 2 (left). Selected residues predicted to make symmetrical contacts are indicated in the homodimer AlphaFold model (right). (**C**) Exponentially growing cultures expressing AuxB mutants with single cysteine residues were treated with copper-phenanthroline. FLAG-AuxB monomer and dimer species were detected by FLAG Western blot. (**D**) Coomassie-stained PAGE gel of co-immunoprecipitation of AuxB^WT^ and AuxB^W86A^ as described in Fig. 1D. AuxB^W86A^ pulls down approximately four times the amount of PknB compared to AuxB^WT^. (**E**) UV trace of protein elution from size exclusion chromatography column. FLAG-AuxB or FLAG-AuxB^W86A^ was co-expressed with 6xHis-PknB in *E. coli*. FLAG-AuxB was also expressed alone. Membrane fractions were solubilized, purified by affinity purification over α-FLAG resin, and separated by size exclusion chromatography. Molecular weights of the AuxB-PknB and AuxB-AuxB dimers are indicated. (**F**) Model for AuxB-PknB equilibrium in cells. AuxB can form a homodimer or an AuxB-PknB heterodimer. AuxB^WT^ (top) favors the homodimer conformation. Disrupting the homodimer interface via the Trp86Ala mutation in AuxB^W86A^ (bottom) shifts this equilibrium to favor the AuxB-PknB heterodimer.

We used AlphaFold to predict possible configurations for AuxB and PknB (54, 55). AlphaFold generated high-confidence models for an AuxB homodimer and an AuxB-PknB heterodimer (Fig. 2A). Analysis of the interfaces showed that the AuxB homodimer buries the interface that interacts with PknB, suggesting the homo- and heterodimers are mutually exclusive. To test the AuxB homodimer model, we introduced cysteines at four residues at the predicted interface (Fig. 2B) and treated cells expressing these AuxB variants with copper-phenanthroline to promote disulfide bond formation (56). For the Trp86Cys variant, in which the cysteines were almost perfectly aligned to form a bond in the predicted homodimer structure, we observed a strong cross-link (Fig. 2C), validating the predicted interface and showing that AuxB can exist as a homodimer.

Trp86 is highly conserved in DUF4064 proteins, and the predicted close interaction between tryptophan side chains suggested that this residue stabilizes the homodimer. We therefore hypothesized that replacing Trp86 with Ala would destabilize the AuxB homodimer, which might increase the amount of AuxB-PknB complex. To test this hypothesis, we constructed a strain expressing AuxB^W86A^ to compare with AuxB^WT^. After testing that these strains had similar growth rates (Fig. S2A) and PknB protein abundance (Fig. S2B), we expressed AuxB^W86A^ and immunoprecipitated it. Compared with AuxB^WT^, AuxB^W86A^ pulled down approximately four times more PknB (Fig. 2D; Fig. S2C). Consistent with these results, size exclusion chromatography of purified AuxB^W86A^ and AuxB^WT^ complexes with PknB showed an increase in heterodimer and a decrease in homodimer for AuxB^W86A^ (Fig. 2E). We concluded that the AuxB homodimer and the AuxB-PknB heterodimer exist in an equilibrium that can shift by perturbing stability of the complexes (Fig. 2F).

### AuxB antagonizes PknB through direct association

**Fig 3:**
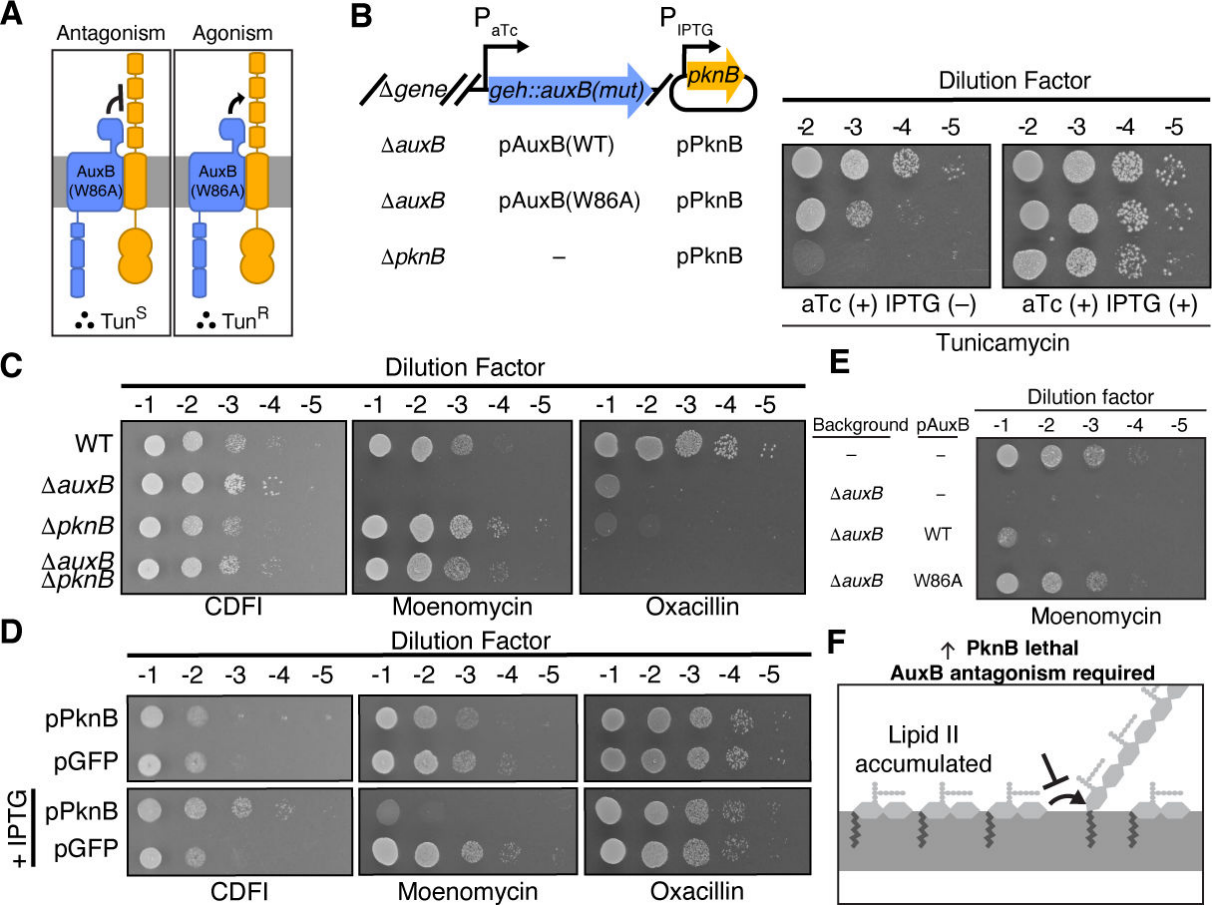
AuxB antagonizes PknB to reduce compound sensitivity. (**A**) Models for the effect of AuxB on PknB, and phenotypes expected with each model. (**B**) Spot assays of the indicated strains plated on tunicamycin (0.4 μg/mL). Cells expressing AuxB^W86A^, which binds more PknB than wild-type AuxB, have a growth defect that is suppressed by overexpressing PknB. (**C**) Spot assays of the indicated strains plated on compounds that accumulate lipid II either in the outer leaflet (moenomycin, 20 ng/mL) or the inner leaflet (CDFI [2-(2-chlorophenyl)-3-(1-[(2,3-dimethylphenyl)methyl]piperidin-4-yl)-5-fluoro-1H-indole], 400 ng/mL) of the membrane, or inhibit transpeptidases that cross-link peptidoglycan (oxacillin, 125 ng/mL). Deletion of *pknB* suppresses Δ*auxB* lethality on the lipid II accumulator, moenomycin. (**D**) Cells containing a multicopy plasmid encoding isopropyl β-d-1-thiogalactopyranoside (IPTG)-inducible PknB or GFP were plated on the same compounds as in (**C**). Cells overexpressing PknB phenocopy Δ*auxB* compound sensitivity. CDFI, 400 ng/mL; moenomycin, 15 ng/mL; oxacillin, 125 ng/mL. (**E**) Δ*auxB* complemented at an ectopic locus with either *auxB^WT^* or *auxB^W86A^* was plated on inducer and moenomycin (15 ng/mL). (**F**) When compounds prevent lipid II usage and cause lipid II to accumulate in the outer leaflet of the membrane, AuxB is required to antagonize PknB.

Knowing that AuxB interacts with PknB, we asked whether AuxB modulates PknB function. It has been shown that strains lacking *pknB* cannot grow on the wall teichoic acid synthesis inhibitor tunicamycin (17, 36). If AuxB binding antagonizes PknB, the strain expressing AuxB^W86A^ should phenocopy a *pknB* hypomorph and be more sensitive to tunicamycin. Alternatively, if AuxB is an agonist of PknB, the AuxB^W86A^ strain should phenocopy a *pknB* hypermorph and be less sensitive to tunicamycin (Fig. 3A). The AuxB^WT^ and AuxB^W86A^ complementation strains grew similarly (Fig. S2E and F). However, when we induced expression of *auxB^W86A^,* the strain became more sensitive to tunicamycin in a spot assay; this sensitivity was suppressed by PknB overexpression (Fig. 3B; Fig. S2D and G). Collectively, these results are consistent with a model wherein AuxB binding antagonizes a PknB function important for growth on tunicamycin.

### Dysregulated PknB can explain Δ*auxB* sensitivity to compounds that inhibit lipid II polymerization

Given that AuxB can antagonize PknB, we asked if we could ascribe some of Δ*auxB*’s compound sensitivities to PknB dysregulation. To test this, we deleted *pknB* from both the WT and Δ*auxB* backgrounds, and we overexpressed PknB in a WT background. After confirming via phosphoproteomics that overexpressed PknB was active (Fig. S2H through K) and was properly localized to the membrane (Fig. S2L), we plated the resulting strains on cell envelope-targeting compounds with diverse mechanisms of action (Fig. 3C and D; Fig. S3A through C). As previously reported, Δ*auxB* is lethal on Congo Red, which inhibits lipoteichoic acid synthase, and on oxacillin, which inhibits cell wall cross-linking but does not affect levels of the cell wall precursor lipid II (Fig. S3D) (38, 61–63). It is also lethal on moenomycin, vancomycin, and daptomycin, three compounds that impair peptidoglycan polymerization and cause accumulation of the peptidoglycan precursor lipid II in the outer leaflet of the membrane (Fig. S3E) (64, 65). Finally, *auxB* deletion was either neutral or beneficial to growth on 2-(2-chlorophenyl)-3-(1-[(2,3-dimethylphenyl)methyl]piperidin-4-yl)-5-fluoro-1H-indole (CDFI), a compound that blocks lipid II translocation from the inner to the outer leaflet of the membrane (Fig. 3C; Fig. S3C) (66–68). We found that deleting *pknB* in a wild-type background had no impact on viability on Congo Red and did not suppress Congo Red lethality in a Δ*auxB* background. Deleting *pknB* similarly did not appear to affect viability on CDFI. On oxacillin, deleting *pknB* was lethal, consistent with previous findings (17, 18, 69). We confirmed that these phenotypes were not due to pleiotropic growth differences between strains because the deletion strains grew at wild-type rates (Fig. S3F). In contrast, for the compounds that caused lipid II accumulation in the outer leaflet of the membrane, deleting *pknB* suppressed Δ*auxB* lethality. For these same compounds, but not for the compounds that target other steps in cell envelope assembly, overexpressing PknB in a WT background was toxic (Fig. 3D; Fig. S3B). Together, our results are consistent with a model in which PknB is dysregulated in Δ*auxB*. If so, increasing the AuxB-bound fraction of PknB using the *auxB^W86A^* allele should result in better growth on lipid II accumulating compounds. When we complemented Δ*auxB* with *auxB^WT^* or *auxB^W86A^,* AuxB^W86A^ supported better growth on compounds that caused lipid II to accumulate in the outer leaflet of the membrane (Fig. 3E; Fig. S3G). We conclude that AuxB antagonizes PknB through direct association, and this antagonism contributes to intrinsic resistance to antibiotics with mechanisms that accumulate lipid II in the outer leaflet of the membrane (Fig. 3F; Fig. S3H).

PASTA kinases are broadly conserved in *Bacillota* and *Actinobacteria*, and we wondered whether deleting *pknB* orthologs from other organisms would also provide a fitness advantage on antibiotics that cause lipid II accumulation in the outer leaflet of the cytoplasmic membrane. We thought this might be the case because PknB overexpression in *Mycobacterium smegmatis* was previously shown to induce sensitivity to vancomycin (70). Indeed, when we deleted *pknB* from the *Mycobacteriales* species *Corynebacterium glutamicum,* the mutant grew better than wild type on vancomycin (Fig. S3I). In contrast, when plated on the β-lactam ampicillin, the *pknB* mutant was unable to grow. We also tested *Bacillus subtilis* where the PknB ortholog is PrkC. Like in the other two organisms, we found that deleting *prkC* from *B. subtilis* improved growth on vancomycin; consistent with previous reports (30, 71, 72), it also increased sensitivity to β-lactam treatment (Fig. S3J). Corroborating our observation in *S. aureus*, PknB is not always beneficial to cells when cell envelope processes are inhibited.

### PknB’s toxicity on inhibitors of lipid II polymerization is due to the PASTA domain

**Fig 4:**
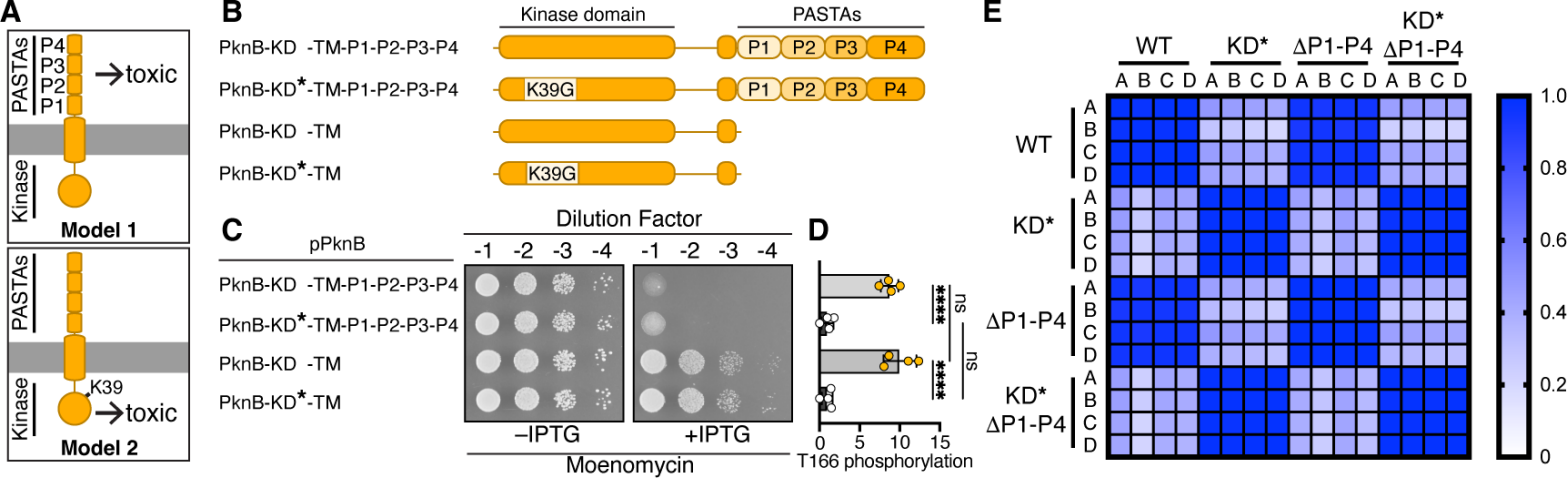
PknB’s PASTA domain potentiates toxicity of peptidoglycan polymerase inhibitors without impacting kinase activity. (**A**) Models for how PknB leads to compound sensitivity on peptidoglycan polymerase inhibitors. Model 1: the PASTA domain causes toxicity. Model 2: kinase signaling causes toxicity. (**B**) Schematic of PknB variants tested in the spot assays in panel C. KD* = Lys39Gly substitution in the kinase domain, which impairs catalytic activity. P1 through P4 refer to the repeats in the PASTA domain. (**C**) Δ*pknB* strains harboring a multicopy plasmid that encodes one of the indicated PknB variants were plated on moenomycin (15 ng/mL). (**D**) Phosphorylation of PknB Thr166, as a proxy for PknB activation, as determined by quantitative phosphoproteomics on cells expressing the variants from panel C. Phosphorylation of PknB^WT^ is normalized to PknB^KD*^, and PknB^ΔP1-P4^ is normalized to PknB^KD*,ΔP1-P4^ for ease of visualization. *n* = 4 biological replicates. Individual values are plotted with mean ± 1 standard deviation. Significance determined by one-way analysis of variance; **** = *P* < .001. A side-by-side comparison between normalized and raw data can be found in Fig. S4D and E. (**E**) Pearson correlation coefficients between each strain were calculated using all detected phosphoproteins. The correlation is high between strains expressing KD^WT^ variants and between strains expressing KD* variants, but is low between strains expressing KD^WT^ and KD* variants. All phosphosites are listed in Table S5.

We next asked what PknB features cause sensitivity to inhibitors that block polymerization of lipid II. PknB has an intracellular kinase domain, a transmembrane helix, and repeating extracellular PASTA modules (Fig. 4A). To test the importance of kinase domain activity and PASTA domain presence for growth on compounds that cause lipid II accumulation, we made a catalytically impaired PknB variant (Lys39Gly, which is defective in ATP binding) (18), and a variant lacking the PASTA domain (ΔP1-P4) (Fig. 4B). We also made a variant lacking both kinase domain activity and the PASTA domain (K39G,ΔP1-P4). These variants expressed at similar levels to PknB^WT^ and were properly localized to the membrane (Fig. S2L), and strains expressing these variants grew at similar rates (Fig. S4A). When we overexpressed these variants and plated on moenomycin or vancomycin, we found that PknB^K39G^ was as toxic as PknB^WT^ (Fig. 4C; Fig. S4C), but PknB^ΔP1-P4^ was viable. The catalytically impaired and truncated variant, PknB^K39G,ΔP1-P4^, resembled the PASTA truncation alone. Because it has been proposed that the PASTA domain is required for kinase domain activation, we needed to know whether the PknB^ΔP1-P4^ variant was indeed catalytically active in order to interpret these results. We therefore performed quantitative phosphoproteomics on the strains expressing each PknB variant. As one measure of PknB activity, we assessed phosphorylation at Thr166, which is in the activation loop of PknB (23). Phosphorylation at this residue stabilizes the active conformer of the kinase domain (73). Both PknB^WT^ and PknB^ΔP1-P4^ showed comparable levels of phosphorylation at T166 (Fig. 4D; Fig. S4D and E); levels were around 10-fold lower for the K39G kinase mutants. Moreover, we found that the phosphoproteomes for samples with wild-type kinase domains were strongly correlated regardless of whether the PASTA domain was present (Fig. 4E). Therefore, PknB’s PASTA domain is not required for kinase domain activity in cells. Knowing this, we conclude that PknB’s PASTA domain sensitizes cells to compounds that trap lipid II in the outer leaflet, and this phenotype is independent of kinase domain activity.

### PknB’s kinase activity and PASTA domain are differentially important on cell envelope-targeting compounds

**Fig 5:**
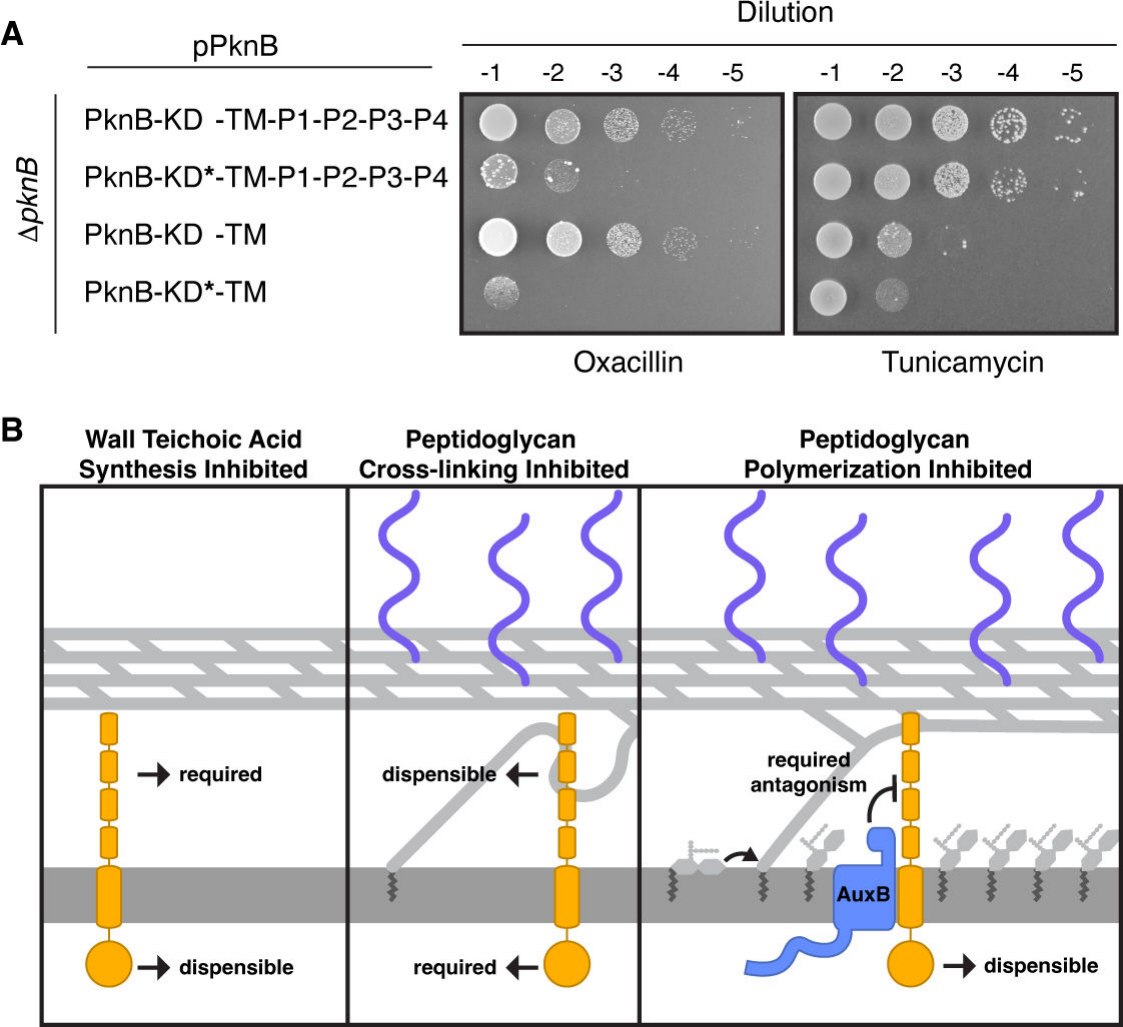
The PASTA domain and kinase activity have non-overlapping roles in cell envelope integrity. (**A**) Exponentially growing cells expressing PknB variants were plated on tunicamycin or oxacillin. PknB’s PASTA domain is required for growth when WTA synthesis is inhibited. Kinase activity does not contribute to growth under this condition. Catalytic activity of PknB’s kinase domain is required when cell wall cross-linking is inhibited. The PASTA domain is not required for growth or catalytic activity under this condition. Tunicamycin, 0.2 μg/mL. Oxacillin, 0.155 μg/mL. (**B**) Summary of PknB’s domain requirements when cell envelope polymers are disrupted. (Left) When cells lack WTAs, PknB’s PASTA domain is required for viability. PknB’s catalytic activity is dispensable and does not contribute to viability when WTA synthesis is inhibited. (Middle) When peptidoglycan cross-linking is inhibited, PknB’s catalytic activity is essential for growth. PknB is catalytically active without its PASTA domain, and the PASTA is dispensable. (Right) Inhibition of peptidoglycan polymerization can cause lipid II to accumulate in the outer leaflet. PknB’s PASTA domain becomes toxic under this condition and requires direct antagonism by AuxB. The kinase activity of PknB does not affect viability when lipid II accumulates in the outer leaflet.

PknB is an IRF to β-lactam antibiotics (17, 18, 69), and kinase domain activity is thought to be required for intrinsic resistance (34, 74, 75). It is also thought kinase activity depends on the PASTA domain. However, the *S. aureus* strain COL encodes a PknB variant that has a stop codon in its first PASTA repeat, and it has been shown that this COL *pknB* allele can complement loss of full-length PknB with respect to intrinsic resistance to β-lactams (34). Using phosphoproteomics, we have now shown that PknB lacking the entire PASTA domain functions as an active kinase in cells. Moreover, this *pknB*^ΔP1-P4^ allele complemented loss of the wild-type allele on oxacillin (Fig. 5A), consistent with results for the truncated *pknB* COL allele. The *pknB^K39G^* alleles, which have impaired catalytic activity, did not complement. We have concluded that PknB’s kinase activity is required for growth when transpeptidation is inhibited, but the PASTA domain is dispensable. Hence, under these conditions, the functions of the two domains are phenotypically separable.

We also tested whether kinase domain activity and PASTA domain presence were required on tunicamycin, another condition where *pknB* is required for viability. Both *pknB^WT^* and *pknB^K39G^* were able to complement Δ*pknB* cells treated with tunicamycin, but neither *pknB^ΔP1-P4^* nor *pknB^K39G,ΔP1-P4^* supported growth under this condition (Fig. 5A). Therefore, when wall teichoic acid synthesis is inhibited, PknB’s PASTA domain is required but its kinase domain activity is not. Collectively, our results show that kinase domain activity and the functions of the PASTA domain are distinct rather than interdependent (Fig. 5B). Taken together with the experiments reported in Fig. 3, our findings also suggest that AuxB antagonizes PknB’s PASTA domain functions.

## DISCUSSION

This work has provided several new insights into the intrinsic antibiotic resistance factors AuxB and PknB. First, we have established that the four-TM helix topology of AuxB places the C-terminal region inside the cell, invalidating the previously proposed model for AuxB function, which relied on the assumption that the C-terminal region of AuxB was extracellular. Second, we have shown that AuxB directly interacts through its intracellular C-terminal region with GpsB, a protein proposed to coordinate activities of cell wall biosynthesis proteins during cell division. Third, we have shown that the membrane domain of AuxB interacts directly with PknB, the only eukaryotic-like serine/threonine kinase in *Staphylococcaceae*. We have also shown that AuxB forms a homodimer in the membrane that is in equilibrium with the AuxB-PknB heterodimer. Fourth, we have presented evidence that AuxB binding antagonizes some of PknB’s functions; we therefore speculate that under some conditions, the equilibrium between the AuxB homodimer and the AuxB-PknB heterodimer shifts to modulate this antagonism. Finally, we have established that requirements for kinase domain activity and the PASTA domain are distinct on compounds that target different steps in cell envelope biogenesis. These findings about PknB are not consistent with the prevailing model in which the main purpose of PknB’s PASTA domain is to bind lipid II to promote kinase domain activation. We therefore suggest that there is an undiscovered role for the PASTA domain in *S. aureus* that is crucial under some conditions (e.g*.,* tunicamycin stress) and toxic under others (e.g*.,* lipid II accumulation in the outer leaflet of the membrane), and that AuxB modulates this function.

PknB was proposed to behave like eukaryotic receptor tyrosine kinases, which are activated by ligand-mediated dimerization. Bivalent ligands bring these receptors into proximity, and the intracellular kinase domains can then activate by trans-autophosphorylation. Lombana et al. reported that *Mycobacterium tuberculosis* PknB activated poorly *in vitro* when it was purified without its membrane helix or PASTA domain; however, fusing the kinase domain to a rapamycin-binding domain and adding rapamycin to force dimerization led to a marked increase in PknB activation (31). This observation inspired the model that PknB, like receptor kinases in eukaryotes, requires a ligand to dimerize. It was later shown that *S. aureus* PknB’s PASTA domain could associate with lipid II, and it was proposed that lipid II is the bivalent ligand that activates PknB (26, 76). However, our studies on *S. aureus* PknB do not align with a model where the PASTA and kinase domains of PknB are interdependent, with lipid II promoting kinase domain activity. First, when cells are treated with compounds that cause lipid II to accumulate outside the cell, PknB’s kinase activity has no impact on survival outcomes. Second, PknB is active *in vivo* without its PASTA domain and still serves as an IRF under β-lactam treatment. Third, PknB’s PASTA domain, but not its kinase domain activity, is required for growth on the wall teichoic acid inhibitor tunicamycin. Collectively, these findings show that lipid II binding to the PASTA domain is not required for kinase domain activity and that the PASTA domain is important under some conditions independent of kinase domain activity. Consistent with our conclusion that lipid II binding is not required for kinase activation, several recent reports have shown that full-length PknB can activate *in vitro* without a ligand (17, 26, 77). Moreover, the Halbedel lab recently showed that PrkA, the PknB ortholog in *Listeria monocytogenes*, is also constitutively active (35).

The finding that PknB’s PASTA domain is required to support growth when WTA synthesis is impaired—even when the kinase domain is inactivated—also implies that the PASTA domain itself has a function separable from kinase domain activity. In *Streptococcus pneumoniae*, the PASTAs are known to interact with the pneumococcal hydrolase LytB to augment its hydrolysis of the cell wall (78, 79). Perhaps the PASTA domain of *S. aureus* PknB plays an analogous role in regulating activity of extracellular proteins. We note that possible suppressors of *pknB*^Δ^*^P1-P4^* are evident on tunicamycin plates (Fig. 5A) and might provide a starting point for future studies of PknB’s PASTA domain. We have also shown that the PASTA domain is toxic on compounds that accumulate lipid II in the outer leaflet of the membrane. Identifying the PASTA domain function that is important when wall teichoic acid synthesis is disrupted might mechanistically explain this observation. Alternatively, the toxicity of the PASTA domain under these conditions could be due to its ability to bind lipid II. If indeed the PASTA domain can bind lipid II in cells as it does *in vitro*, PknB might sequester free lipid II and thereby synergize with compounds that block lipid II polymerization. However, testing the explanation that toxicity is due to lipid II sequestration is not straightforward because amino acid substitutions that disrupt PASTA domain interactions with lipid II would also disrupt interactions with other muropeptide species that may be important for different reasons.

**Fig 6:**
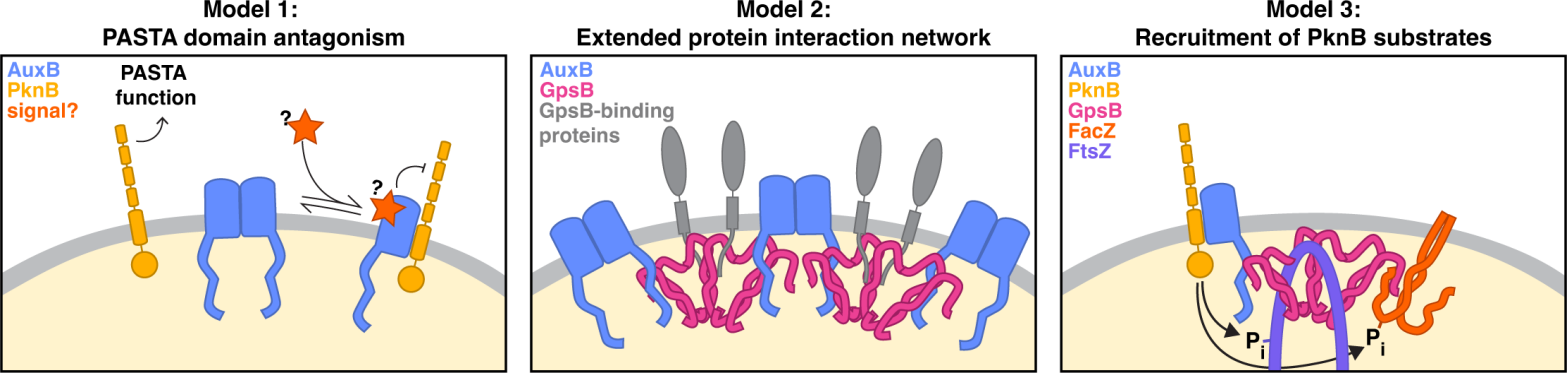
Working models for roles of the AuxB and its interactors. Model 1: AuxB antagonizes a function of PknB’s PASTA domain. AuxB exists in equilibrium between a homodimer and an AuxB-PknB heterodimer. In the heterodimer, which may increase in response to a signal, AuxB antagonizes the PASTA domain. Model 2: AuxB and GpsB may form extended protein interaction networks that coordinate GpsB-binding partners. GpsB forms a hexamer that can directly interact with up to six other proteins. Interactions between GpsB and AuxB would allow extended protein interaction networks. Model 3: AuxB links GpsB-binding proteins to PknB’s kinase domain for phosphorylation. AuxB interacts with GpsB and PknB, and several of GpsB’s binding partners are PknB substrates. An AuxB:PknB:GpsB:GpsB-binding-protein complex may present cell division proteins to PknB for phosphorylation.

We have shown that AuxB can antagonize PknB and that hyper-antagonism is harmful on tunicamycin, but helpful on compounds like moenomycin that cause lipid II to accumulate in the membrane. Given the importance of the PASTA domain under both sets of conditions, AuxB evidently antagonizes a PASTA domain function. We therefore speculate that AuxB plays a physiological role in modulating interactions of the PASTA domain with other factors in the cell. Because perturbing the stability of the AuxB homodimer interface causes an increase in AuxB-PknB heterodimer, we posit that there may be a cellular signal that shifts the equilibrium between AuxB homodimer and AuxB-PknB heterodimer. In this regard, the loose resemblance between AuxB’s transmembrane domain and eukaryotic tetraspanin proteins is worth noting. Eukaryotic tetraspanins form protein-protein interactions that are dependent on lipid signals (50, 51). Because both WTA precursors and lipid II are synthesized on an undecaprenyl phosphate carrier, an undecaprenyl metabolite is an attractive candidate to consider as a signal for AuxB (Fig. 6, model 1). An additional function of eukaryotic tetraspanins is to establish extended protein-protein interactions and lipid microdomains in eukaryotic membranes (52, 56, 80). It is possible that AuxB performs an analogous role in *S. aureus*. AuxB homodimerizes, and its C-terminus SRQAKY motif can interact with GpsB, which is a hexamer (81, 82). One GpsB hexamer is limited to six interacting proteins, but repeated AuxB-GpsB interactions might cluster GpsB hexamers to facilitate extended protein complexes that coordinate functions of cell wall assembly machinery (Fig. 6, model 2). Alternatively, because AuxB binds both PknB and GpsB using different domains, it could bring these proteins together. In other organisms, GpsB is thought to augment PknB activity through direct association. This has not been demonstrated in *S. aureus*. Our findings suggest that AuxB may bring GpsB and PknB into proximity for phosphorylation of GpsB and GpsB-binding partners. In support of this hypothesis, we have observed that some PknB substrates identified in our data (Tables S4 and S5) and elsewhere (83), including FacZ and FtsZ (Fig. S5), are also GpsB-binding proteins (Fig. 6, model 3).

Although there are still unanswered questions about the functions of AuxB, as there are about PknB and GpsB, the direct interactions between these three proteins, combined with the observation that AuxB can exist as a homodimer and a competing AuxB-PknB heterodimer, likely contribute to the importance of AuxB as an IRF. Beyond that, we conclude that AuxB, through its physical interactions with GpsB and PknB, helps coordinate cell wall biogenesis with cell division (Fig. 6).

## MATERIALS AND METHODS

### Materials

All reagents were purchased from Sigma-Aldrich unless otherwise specified. Recombinant lysostaphin was obtained from AMBI Products LLC. Reducing and non-reducing Laemmli SDS-Sample Buffer (6× working concentration for each) were purchased from Boston BioProducts. Tryptic soy broth (TSB) powder, lysogeny broth (LB) powder, brain heart infusion (BHI), and Bacto agar were purchased from Becton Dickinson. Terrific broth (TB) powder was purchased from VWR. Kanamycin, carbenicillin, daptomycin, and isopropyl β-d-1-thiogalactopyranoside (IPTG) were purchased from Gold Biotechnology. CDFI was purchased from Enamine. Tris-Glycine (TGX) SDS-PAGE gels, TGX running buffer, Blotting Grade Blocker Nonfat Dry Milk, immunoblot polyvinylidene fluoride (PVDF), and 0.2 μm pore nitrocellulose were purchased from Bio-Rad. Anhydrotetracycline (aTc) and sodium (2-sulfonatoethyl)methanethiosulfonate (MTSES) were purchased from Cayman Chemical. Imperial Protein Stain, Superblock T20 (TBS), Pierce ECL Western Blotting Substrate, Immobilon-FL PVDF membrane, Pierce streptavidin-horseradish peroxidase (HRP), and Pierce High-Select Fe-NTA Phosphopeptide Enrichment kit were purchased from Thermo Fisher. Amersham ECL Prime Western Blotting Detection Reagent was purchased from GE. BioMax Light Film was purchased from Kodak. n-Dodecyl-β-d-maltoside (DDM) was purchased from Avanti Polar Lipids. G1-α-Flag resin and 1×FLAG peptide were purchased from GenScript. DNA polymerases for PCR (Q5 and Phusion), restriction enzymes, T4 ligase and buffer, and KLD master mix were purchased from New England Biolabs. KOD polymerase was purchased from EMD Millipore. In-Fusion HD Cloning Mix was purchased from Takara Bio. Oligos used for cloning were purchased from Integrated DNA Technologies. Buffers for DNA plasmid and PCR purification were purchased from Qiagen. Vancomycin-HCl was obtained from Merck. Moenomycin A was purified from Flavomycin as described (84) and provided by Daniel Kahne.

### SCAM to determine protein topology

SCAM protocols were modified from Butler et al*.* (48). Strains were streaked onto tryptic soy agar (TSA; tryptic soy broth supplemented with 1.5% agar), and overnight cultures were inoculated with single colonies into TSB. Subcultures were initiated by a 1:100 (vol/vol) dilution into 50 mL TSB supplemented with 0.5 mM IPTG and grown to OD_600_ = 0.8–1.0. Cultures were pelleted (5,000 *g*, 4°C, 5 min), and the supernatant was removed. The pellet was resuspended in wash buffer (phosphate buffered saline [1×PBS], pH 7.4) and pelleted (5,000 *g*, 4°C, 5 min). Cells were resuspended to OD_600_ = 100 in protoplasting buffer (1× PBS, 250 mM sucrose, 1.5 μg/mL lysostaphin, 67 U/mL benzonase, pH 7.4) and rotated head-over-tail at 37°C for 1 h. Protoplasted cells were separated into four aliquots of 50 μL each. Two aliquots were left untreated at room temperature for 1 h, and the other two were rocked head-over-tail for 1 h at room temperature with either 5 mM of MTSES or 5 mM of N-ethylmaleimide (NEM). After incubation, 500 μL protoplasting buffer base (1× PBS, 250 mM sucrose) was added to all samples. All samples were pelleted (5,000 *g*, 4°C, 5 min), and supernatants were removed. Samples were resuspended once with 500 μL of protoplasting buffer base before pelleting once more (5,000 *g*, 4°C, 5 min). Samples were resuspended in 50 μL of lysis buffer (15 mM Tris-HCl, 1% wt/vol SDS, 6 M urea, pH 7.4). Samples were mixed with methoxypolyethylene glycol maleimide (mal-PEG, M_n_ = 5,000) to a final concentration of 5 mM mal-PEG. Samples were rotated head-over-tail for 1 h in the dark. The reaction was quenched with 2× Buffer AB (6.84 mM Na_2_HPO_4_, 3.16 mM NaH_2_PO_4_, 50 mM Tris-HCl, pH 6.8, 6 M urea, 1% vol/vol β-mercaptoethanol, 3% wt/vol SDS, 10% vol/vol glycerol, 0.1% wt/vol bromophenol blue). Samples were centrifuged (21,000 *g*, 2 min) to pellet DNA, and the supernatants were loaded onto a polyacrylamide 4-20% TGX SDS-PAGE gel and run in TGX buffer (25 mM Tris, 192 mM glycine, 0.1% wt/vol SDS, pH 8.3) at 180V for 45 min. The gel was transferred onto a nitrocellulose membrane using a Transblot Turbo system using the preset Mixed MW setting. The membrane was blocked with blocking buffer (5% wt/vol milk, 1× TBS, 0.1% vol/vol Tween 20, pH 7.5) for 1 h at room temperature. A 1:2,000 (vol/vol) dilution of HRP-conjugated α-FLAG M2 was added directly to the blocking buffer and equilibrated for 1 h at room temperature. The membrane was washed three times with wash buffer (1× TBS, 0.1% vol/vol Tween 20, pH 7.5) for 10 min each wash. The blot was developed using Pierce ECL Western Blotting Substrate and imaged on a Bio-Rad ChemiDoc Imager using the preset chemiluminescence program.

### Co-immunoprecipitation (Co-IP) of FLAG-tagged proteins from *S. aureus*

Co-IP protocols were modified from Bartlett et al*.* (42). *S. aureus* strains were streaked to isolate single colonies on TSA. Single colonies were used to start 12.5 mL cultures in TSB. Cultures were grown overnight at 30°C with shaking. Overnight cultures were diluted 1:100 (vol/vol) into 1 L of TSB supplemented with 0.4 µM aTc. Cultures were grown at 37°C with aeration via shaking for 7 h. OD_600_ was measured at collection to ensure that cell mass between strains was comparable. Induced cultures were pelleted (5,000 *g*, 10 min, 4°C), and the supernatant was removed. Cell pellets were resuspended in 30 mL 1× PBS, pH 7.4, and pelleted again (5,000 *g*, 5 min, 4°C). Supernatant was removed and pellets were frozen at −80°C until use. The day of the Co-IP, pellets were suspended in 30 mL lysis buffer (1× PBS, pH 7.4 with 100 μg/mL lysostaphin, 1 EDTA-free cOmplete protease inhibitor cocktail tablet [Roche] per 50 mL liquid total, 0.5 μL benzonase per 50 mL liquid total). The suspension was tumbled head-over-tail at 37°C for 1 h to begin lysis. The resuspension was cooled on ice for 15 min before lysis was completed by passaging through an EmulsiFlex-C3 cell disruptor (Avestin) five times at 15,000–20,000 psi. Insoluble debris was removed by centrifugation (10,000 *g*, 15 min, 4°C), and membranes were collected from the supernatant by ultracentrifugation (140,000 *g*, 45 min, 4°C). Membrane pellet was resuspended in 3 mL of membrane extraction buffer (1× PBS, pH 7.4, 400 mM NaCl, 1% wt/vol DDM) using a Dounce tissue grinder (Wheaton). Membrane extraction was completed by head-over-tail tumbling of the mixture at 4°C for 1 h. The membrane homogenate was then diluted with 3 mL of a “no salt” buffer (1× PBS, pH 7.4, 1% wt/vol DDM). A total of 37.5 µL (packed volume) of pre-equilibrated α-FLAG M2 magnetic beads was then added to the membrane homogenate and equilibrated at 4°C with tumbling for 1 h. Beads were collected from the liquid using a magnetic tube rack. The beads were washed four times in 500 μL of wash buffers containing a decreasing gradient of DDM (5 min each at 4°C; all buffers contain 1× PBS, pH 7.4, 133 mM additional NaCl; 0.5% wt/vol DDM for the first wash, 0.25% wt/vol DDM for the second wash, 0.125% wt/vol DDM for the third wash, and 0.0625% wt/vol DDM for the fourth wash) and separated from the wash buffer after each wash by collection on the magnetic rack. Proteins were eluted from the beads in 150 μL elution buffer (1× PBS, pH 7.4, 133 mM NaCl, 150 μg/mL 3×FLAG peptide, and 0.05% wt/vol DDM) with tumbling (30 min, room temperature). The elution fraction was separated from the beads using a magnetic collection rack. Elution fractions were mixed with 6× reducing Laemmli Buffer and loaded onto a polyacrylamide TGX SDS-PAGE gel and run in TGX buffer at 180V for 30 min. The gel was then stained with Imperial Protein Stain at room temperature for 2 h and de-stained overnight with MilliQ water. Bands were excised and submitted to the Taplin Mass Spectrometry Facility at Harvard Medical School for identification.

### Expression and purification of *S. aureus* AuxB, AuxB^W86A^, and PknB

A previously described protocol was adopted with minor modifications (85). All media used for growth of expression strains was supplemented with 100 μg/mL carbenicillin. Overnights were initiated for AGS046 (*E. coli* KRX encoding FLAG-AuxB) and AGS045 (*E. coli* KRX encoding FLAG-AuxB and His_6_-PknB) from 5 to 10 colonies streaked out from glycerol stocks. An overnight was initiated from 5 to 10 colonies of C43 freshly transformed with pTS090 (encoding FLAG-AuxB^W86A^ and His_6_-PknB). Overnights were subcultured 1:100 (vol/vol) in 1 L TB at 37°C with shaking until OD_600_ = 0.7–0.8. Protein expression was induced by adding 500 μM IPTG. For KRX strains (AGS045 and AGS046), 2 mM L-rhamnose was also supplemented. Cells were grown in the presence of inducer for 5 h at 37°C and then harvested by centrifugation (4,200 *g*, 15 min, 4°C). Cells were resuspended in 50 mL lysis buffer (50 mM HEPES, pH 7.5, 150 mM NaCl, 1 mg/mL lysozyme, 250 μg/mL DNase, 2 mM MgCl_2_, and one cOmplete protease inhibitor tablet) and lysed by passing the resuspended cells through an EmulsiFlex-C3 cell disruptor three times at 15,000 psi. Cell debris was removed by centrifugation (12,000 *g*, 5 min, 4°C), and the membrane fraction was collected by ultracentrifugation of the supernatant (100,000 *g*, 1 h, 4°C). The membrane pellet was resuspended in solubilization buffer (50 mM HEPES, pH 7.5, 0.5 M NaCl, 1% wt/vol DDM, 10% vol/vol glycerol) using a Dounce tissue grinder. The resulting mixture was rocked for 1.5 h at 4°C before ultracentrifugation (100,000 *g*, 30 min, 4°C). The supernatant containing the DDM-solubilized protein was supplemented with 2 mM CaCl_2_ and loaded onto a G1-α-Flag antibody resin. The resin was washed three times with 20 mL of buffer A (50 mM HEPES, pH 7.5, 0.5 M NaCl, 0.05% wt/vol DDM, 10% vol/vol glycerol) supplemented with 2 mM CaCl_2_, and the bound protein was eluted from the column with 10 mL of buffer A supplemented with 5 mM EDTA and 0.2 mg/mL FLAG peptide. The eluate was further purified by size exclusion chromatography with a Superdex 200 Increase 10/300 GL column equilibrated in buffer A. Fractions containing the desired protein were concentrated by centrifugal filtration and the concentrated protein was stored at −80°C.

### AlphaFold/ColabFold prediction of protein complexes

ColabFold was used to generate AlphaFold-based complexes (54, 55). For the AuxB homodimer and the AuxB-PknB heterodimer, the MMseqs2 option was used for the multiple sequence alignment with only unpaired sequences. The “subsample_msa” option and “use_turbo” options were selected. Three recycles were used. Protein complexes were visualized with ChimeraX.

### Cysteine disulfide cross-linking and Western blotting

*In situ* oxidation of disulfides was adapted from a previously reported protocol (56). Overnight cultures were inoculated with single colonies in TSB and grown at 30°C with aeration by shaking. Bacteria were diluted to OD_600_ = 0.01 in TSB supplemented with 0.4 μM aTc and grown at 30°C to OD_600_ = 0.5. Cells were collected from 1.5 mL of culture by centrifugation (5,000 *g*, 1 min), resuspended in 500 μL of reaction buffer (50 mM HEPES, 150 mM NaCl, 0.6 mM CuSO_4_, 1.8 mM phenanthroline), and incubated at room temperature (15 min). After incubation, cells were collected by centrifugation (5,000 *g*, 1 min) and washed with quenching buffer (50 mM HEPES, 150 mM NaCl, 10 mM NEM) twice. Cells were collected once more by centrifugation (5,000 *g*, 1 min), snap frozen in liquid nitrogen, and stored at −80°C until use.

Dimerization was determined by an immunofluorescent Western blot modified from a previously described protocol (42). Pellets from copper-phenanthroline-treated cultures were lysed in lysis buffer (1× HEPES, 100 μg/mL lysostaphin, 1× cOmplete protease inhibitor, benzonase, pH 7.5) for 1 h at 37°C. Lysates were mixed with 6× non-reducing Laemmli SDS-Sample Buffer. Samples were loaded onto a polyacrylamide TGX SDS-PAGE gel and run in TGX buffer at 150V for 90 min. The gel was transferred to Immobilon-FL PVDF membrane using a Transblot Turbo System with the preset Mixed MW setting. The membrane was blocked with Superblock T20 (TBS) for 1 h at room temperature. The membrane was then incubated with a 1:1,000 (vol/vol) dilution of α-FLAG M2 produced in mouse (Sigma, F1804) for 1 h at room temperature. After equilibration, the membrane was washed three times with 1× TBS supplemented with 0.1% vol/vol Tween 20 (5 min each wash). The membrane was then equilibrated for 1 h at room temperature in Superblock T20 (TBS) containing a 1:20,000 (vol/vol) dilution of α-mouse-AlexaFluor488 (Abcam, ab150113). The secondary-adsorbed membrane was washed three times with 1× TBS supplemented with 0.1% vol/vol Tween 20 (5 min each wash) and then five times with 1× TBS (5 min each) to remove residual Tween. The membrane was imaged on a Bio-Rad ChemiDoc using the preset AlexFluor488 program.

### Growth curves

Overnight cultures were inoculated with single colonies in TSB and grown at 30°C with aeration by shaking. Bacteria were diluted 1:500 in 3 mL media and grown to OD_600_ ~ 0.3-0.5 (TSB ± 0.4 μM aTc at 30°C for Fig. S2A; TSB + 2.5 μg/mL erythromycin ± 1 mM IPTG at 37°C for Fig. S2E; TSB + 2.5 μg/mL erythromycin ± 0.4 μM aTc at 37°C for Fig. S2F; TSB at 30°C for Fig. S3F; TSB + 2.5 μg/mL erythromycin ± 1 mM IPTG at 37°C for Fig. S4A). Bacterial cultures that lacked inducer during subculture were diluted to OD_600_ = 0.1 in fresh media that also lacked inducer, and cultures that contained inducer during subculture were diluted to OD_600_ = 0.1 in fresh media that contained inducer. OD_600_ normalized cultures were aliquoted (150 μL) in a clear flat-bottom 96-well plate. Cultures were grown in a SpectraMax M2 microplate reader at 30°C or 37°C as indicated above. OD_600_ measurements were taken every 10 min, and the plate was shaken continuously between readings.

### Western blotting of PknB-FLAG with various AuxB variants

Overnight cultures were inoculated with single colonies in TSB and grown at 30°C with aeration by shaking. Bacteria were diluted 1:500 in TSB ± 0.4 μM aTc and grown at 30°C to OD_600_ ~1.0. Cells were collected from 1.5 mL of culture by centrifugation (5,000 *g*, 1 min). Cells were resuspended to OD_600_ = 10 in lysis buffer (1× PBS, 1× cOmplete EDTA-free protease inhibitor, 100 μg/mL lysostaphin, 1 μL benzonase per 50 mL buffer) and incubated at 37°C for 1 h. Samples were mixed with 6× Laemmli Buffer, loaded onto a polyacrylamide 4-20% TGX SDS-PAGE gel, and run in TGX buffer at 180 V for 45 min. The gel was transferred to Immobilon-FL PVDF membrane using a Transblot Turbo System with the preset Mixed MW setting. The membrane was blocked with Superblock T20 (TBS) with 10 μg/mL IgG from human serum (Sigma, I4506) for 1 h at room temperature. The membrane was then incubated with a 1:2,000 (vol/vol) dilution of α-FLAG M2 produced in mouse (Sigma, F1804) for 1 h at room temperature. After equilibration, the membrane was washed three times with 1× TBS supplemented with 0.1% vol/vol Tween 20 (5 min each wash). The membrane was then equilibrated for 1 h at room temperature in Superblock T20 (TBS) containing 10 μg/mL IgG from human serum and a 1:20,000 (vol/vol) dilution of α-mouse-AlexaFluor488 (Abcam, ab150113). The secondary-adsorbed membrane was washed three times with 1× TBS supplemented with 0.1% vol/vol Tween 20 (5 min each wash) and then five times with 1× TBS (5 min each) to remove residual Tween. The membrane was imaged on a Bio-Rad ChemiDoc using the preset AlexFluor488 program.

### PknB overexpression and Western blotting

Overnight cultures were inoculated with single colonies in TSB and grown at 30°C with aeration by shaking. Bacteria were diluted to OD_600_ = 0.01 in TSB supplemented with 1 mM IPTG and grown at 30°C to OD_600_ ~0.3. Cells were collected from 1.5 mL of culture by centrifugation (5,000 *g*, 1 min). Cells were resuspended to OD_600_ = 1.5 in lysis buffer (1× PBS, 1× cOmplete EDTA-free protease inhibitor, 100 μg/mL lysostaphin, 1 μL benzonase per 50 mL buffer) and incubated at 37°C for 15 min. A total of 50 μL was reserved as the “lysate” fraction and mixed with 10 μL 6× Laemmli Buffer. The membrane fraction was pelleted by ultracentrifugation (100,000 *g*, 30 min, 4°C) from 200 μL of the remaining lysate fraction. A total of 50 μL of ultracentrifugation supernatant was reserved as the “cytosol” fraction and mixed with 10 μL 6× Laemmli Buffer. The remaining supernatant was discarded. The membrane was resuspended in 200 μL 1× Laemmli Buffer and was used as the “membrane” fraction.

Samples were loaded onto a polyacrylamide 4-20% TGX SDS-PAGE gel and run in TGX buffer at 180 V for 45 min. The gel was transferred to Immobilon-FL PVDF membrane using a Transblot Turbo System with the preset Mixed MW setting. The membrane was blocked with Superblock T20 (TBS) for 1 h at room temperature. The membrane was then incubated with a 1:2,000 (vol/vol) dilution of α-Myc produced in rabbit (Cell Signaling Technologies, mAb #2278) for 1 h at room temperature. After equilibration, the membrane was washed three times with 1× TBS supplemented with 0.1% vol/vol Tween 20 (5 min each wash). The membrane was then equilibrated for 1 h at room temperature in Superblock T20 (TBS) containing a 1:20,000 (vol/vol) dilution of α-rabbit-AlexaFluor647 (Invitrogen, A-21245). The secondary-adsorbed membrane was washed three times with 1× TBS supplemented with 0.1% vol/vol Tween 20 (5 min each wash) and then five times with 1× TBS (5 min each) to remove residual Tween. The membrane was imaged on a Bio-Rad ChemiDoc using the Coomassie program.

### *S. aureus* spot titers

The following protocol was used for plating on all compounds except the tunicamycin assay shown in Fig. 3B. Overnights were inoculated with single colonies into TSB containing appropriate antibiotics and grown at 30°C. Overnight cultures were back diluted to OD_600_ = 0.01 and cultured at 30°C to OD_600_ = 0.3–0.5. Cultures were normalized to OD_600_ = 0.1, corresponding to “Dilution Factor 1.” Ten-fold serial dilutions were made and plated onto TSA plates containing appropriate selective compounds with or without inducers.

The following protocol was used for plating on tunicamycin in Fig. 3B. Overnights were inoculated with single colonies into TSB containing appropriate antibiotics and grown at 30°C. Overnight cultures were normalized to OD_600_ = 0.1, corresponding to “Dilution Factor 1”. Ten-fold serial dilutions were made and plated onto TSA plates containing appropriate selective compounds with or without inducers.

Concentrations of compounds are indicated in the figure captions describing the corresponding plate. Plates containing vancomycin, moenomycin, daptomycin, or oxacillin were grown at 30°C. The CDFI plates were grown at 30°C, except for the CDFI plate from Fig. S3C, which was grown at 37°C. The tunicamycin plates and the Congo Red plates were grown at 37°C.

### *C. glutamicum* spot titers

Strains were streaked onto BHIS agar (BHI supplemented with 9.1% sorbitol and 1.5% Bacto agar) and grown overnight at 30°C. Overnights were inoculated with single colonies into BHIS and grown at 30°C. To assess sensitivity to vancomycin, subcultures were initiated by diluting 1:200 into fresh BHIS and grown to OD_600_ ~0.2–0.4. Cultures were normalized to OD_600_ = 0.1, corresponding to “Dilution Factor 1”. Ten-fold serial dilutions were made and plated onto BHI plates with and without vancomycin. To assess sensitivity to ampicillin, overnight cultures were normalized to OD_600_ = 0.6, corresponding to “Dilution Factor 0.” Ten-fold serial dilutions were made. Culture volume plated was adjusted to ensure that plating density was the same as in the vancomycin experiment. Cultures were plated onto BHI plates with and without ampicillin. Spots were grown overnight at 30°C before being imaged.

### *B. subtilis* spot titers

Strains were streaked onto LB plates and grown overnight at 37°C. Cultures were inoculated the morning of antibiotic testing by adding one colony to LB media. Cells were grown to mid-logarithmic growth at 37°C with aeration. Cultures were back diluted 20× and grown again to OD_600_ ~0.2–0.4. Cultures were normalized to OD_600_ = 0.1, corresponding to “Dilution Factor 1.” Ten-fold serial dilutions were made and plated onto LB plates with and without drug. Spots were grown overnight at 37°C before being imaged.

### Quantitative phosphoproteomics

To assess wild-type PknB only, independent cultures were inoculated into TSB and grown overnight at 30°C with aeration by shaking. Subcultures were initiated by diluting overnight cultures to OD_600_ = 0.01 in 50 mL fresh media (TSB with 2.5 μg/mL erythromycin) and grown at 30°C to OD_600_ ~0.3. Cultures were split into two to make paired induced and uninduced samples. Both were normalized to 25 mL culture at OD_600_ = 0.2. For the induced sample, 1 mM IPTG was added to induce PknB expression. Cultures were grown at 30°C for 1 additional hour. Cells were collected by centrifugation (10 mL culture, 3,220 *g*, 10 min), and the supernatant was removed. Cell pellets were frozen in liquid nitrogen and stored at −80°C until use.

To assess the full length, PASTA truncated, active, and inactive variants, independent cultures were inoculated into TSB and grown overnight at 30°C with aeration by shaking. Subcultures were initiated by diluting overnight cultures 1:500 in 10 mL fresh media (TSB with 2.5 μg/mL erythromycin, 250 μM IPTG) and grown at 30°C to OD_600_ ~0.3. Cultures were normalized to 10 mL culture at OD_600_ = 0.01. Cultures were grown at 30°C to OD_600_ ~0.6. Cells were collected by centrifugation (10 mL culture, 3,220 *g*, 10 min), and the supernatant was removed. Cell pellets were frozen in liquid nitrogen and stored at −80°C until use.

On the day of (phospho)protein preparation, pellets were resuspended in 1 mL lysis buffer (1× PBS, 100 μg/mL lysostaphin, 1× Halt phosphatase and protease inhibitor, 1× PhosStop phosphatase inhibitor, one cOmplete protease inhibitor tablet per 50 mL buffer, 1 μL benzonase per 20 mL buffer, pH 7.4) and incubated at 37°C for 1 h. Lysis and DNA shearing were completed by extrusion through a 21-gauge needle 5 to 10 times. Lysates were clarified by ultracentrifugation (100,000 *g*, 30 min, 4°C). The soluble fraction was removed and reserved on ice. The membrane pellet was resuspended in 300 μL membrane extraction buffer (1× PBS, 1% wt/vol DDM, 400 mM NaCl, 1× Halt phosphatase and protease inhibitor, pH 7.4) and tumbled head-over-tail for 1 h at 4°C. The solubilized membrane fraction was pooled with the reserved soluble fraction to generate a combined protein solution. Proteins were precipitated from 1 volume of the combined protein solution (150 μL) by adding 4 volumes of methanol (600 μL), 1 volume of chloroform (150 μL), and 3 volumes of water (450 μL), mixing briefly between addition of each liquid. Precipitated protein was pelleted (14,000*g,* 1 min), and the methanol/chloroform/water mixture was removed. The precipitated protein was washed with 4 volumes of methanol (600 μL). The washed protein was collected by centrifugation (21,000 *g*, 2 min), and methanol was removed.

Protein was digested using Lys-C overnight at room temperature followed by trypsin for 6 h at 37°C, both at a 100:1 protein:protease ratio. After digestion, the samples were labeled using the TMTpro16 reagents for 90 min, and the reactions were quenched using hydroxylamine (final concentration of 0.3% vol/vol). The samples were combined equally and subsequently desalted.

Phosphopeptides were enriched from the pooled TMT-labeled mixtures using the Pierce High-Select Fe-NTA Phosphopeptide Enrichment kit (“mini-phos”) (86, 87) following manufacturer’s instructions. The unbound fraction was retained and fractionated using basic pH reversed-phase high-performance liquid chromatography (HPLC). Ninety-six fractions were collected and then consolidated into 12, which were analyzed by liquid chromatography-mass spectrometry (LC-MS) (88).

### Mass spectrometric data collection

Mass spectrometry data were collected using an Orbitrap Astral mass spectrometer (Thermo Fisher Scientific, San Jose, CA) coupled with a Neo Vanquish liquid chromatograph. We used the Orbitrap detector for MS2 analysis. Peptides were separated on a 110 cm µPAC C18 column (Thermo Fisher Scientific). For each analysis, we loaded ~0.5 µg onto the column. Data were acquired for 75 min per fraction (*n* = 12) for whole proteome data and 150 min for the phosphoproteomics data (*n* = 2 injections). The scan sequence began with an MS1 spectrum (Orbitrap analysis, resolution 60,000, 350–1,350 Th, automatic gain control [AGC] target is set to 100%, maximum injection time is set to “auto”). The hrMS2 stage consisted of fragmentation by higher energy collisional dissociation (HCD, normalized collision energy 35%) and analysis using the Orbitrap (AGC 200%, maximum injection time 25 ms, isolation window 0.4 Th, resolution 45,000). Data were acquired using the FAIMSpro interface, the dispersion voltage (DV) was set to 5,000V, the compensation voltages (CVs) were set at −35V, −45V, −55V, −60V, and −70V. The TopSpeed parameter was set at 1 s per CV (Table S4).

### Mass spectrometric data collection

Mass spectrometric data were collected on an Orbitrap Eclipse mass spectrometer coupled to a Vanquish Neo UHPLC. Approximately 1 µg of peptide was separated at a flow rate of 450 nL/min on a 100 µm capillary column that was packed with 35 cm of Accucore 150 resin (2.6 µm, 150 Å; Thermo Fisher Scientific). The scan sequence began with an MS1 spectrum (Orbitrap analysis, resolution 60,000, 350–1350 Th, AGC target is set to 100%, maximum injection time set to “auto”). Data were acquired for 90 min per fraction (*n* = 24) for whole proteome data and 150 min for the phosphoproteomics data (*n* = 2 injections). The hrMS2 stage consisted of fragmentation by HCD (normalized collision energy 36%) and analysis using the Orbitrap (AGC 200%, maximum injection time of at least 120 ms, isolation window 0.6 Th, resolution 30,000 Turbo TMT). Data were acquired using the FAIMSpro interface, the DV was set to 5,000V, the CVs were set at −30V, −50V, and −60V or −40V, −60V, and −70V. The TopSpeed parameter was set at 1 sec per CV (Table S5).

### Data analysis

Spectra were converted to mzXML via MSconvert. The database was concatenated with one composed of all protein sequences for that database in the reversed order. Searches were performed using a 50 ppm precursor ion tolerance for total protein level profiling. The product ion tolerance was set to 0.03 Da. These wide mass tolerance windows were chosen to maximize sensitivity in conjunction with Comet searches and linear discriminant analysis. TMTpro labels on lysine residues and peptide N-termini (+304.207 Da), as well as carbamidomethylation of cysteine residues (+57.021 Da), were set as static modifications, while oxidation of methionine residues (+15.995 Da) was set as a variable modification. In addition, phosphorylation (+79.966 Da) at serine, threonine, and tyrosine residues was also set as variable modifications for phosphopeptide enrichment. Peptide-spectrum matches (PSMs) were adjusted to a 1% false discovery rate (FDR). PSM filtering was performed using a linear discriminant analysis, as described previously and then assembled further to a final protein-level FDR of 1%. Proteins were quantified by summing reporter ion counts across all matching PSMs, also as described previously. Reporter ion intensities were adjusted to correct for the isotopic impurities of the different TMTpro reagents according to manufacturer specifications. The signal-to-noise (S/N) measurements of peptides assigned to each protein were summed, and these values were normalized so that the sum of the signal for all proteins in each channel was equivalent to account for equal protein loading. S/N thresholds were 100 for Eclipse-acquired data and 1,000 for data acquired with the Astral mass spectrometer. Also, Astral-acquired data required a resolution of >45,000. Finally, each protein abundance measurement was scaled, such that the summed signal-to-noise for that protein across all channels equals 100, thereby generating a relative abundance measurement. The mass spectrometry proteomics data have been deposited to the ProteomeXchange Consortium via the PRIDE (89) partner repository with the data set identifier PXD065790.

Significance for protein abundance and phosphosite abundance in Table S4 was determined in GraphPad Prism using multiple paired *t*-tests. Q-values were calculated using a two-stage step-up method of Benjamini, Krieger, and Yekutieli with a desired FDR set to 1%. Values were considered significant if q < 0.05 and if the ratio of induced/uninduced was ≥1.5.

Significance for protein abundance and phosphosite abundance in Table S5 was determined using a script written in R-studio, which has been deposited on GitHub (repository Sisley_AuxB on SuzanneWalkerLab). Each protein and phosphosite was assessed independently using a one-way analysis of variance (ANOVA) with a Tukey’s *post hoc* analysis. Each (phospho)protein was assessed independently using a one-way ANOVA to determine significant strain-to-strain differences. Values were considered significant if the Tukey’s *post hoc* returned *P* < 0.05.

### Determination of lipid II levels under drug treatment

Analysis of lipid II content in the cell was performed as previously described (68). Overnight cultures of *S. aureus* RN4220 were subcultured to OD_600_ = 0.1 and grown to OD_600_ = 0.4–0.5 at 37°C. Two milliliters of the culture was then treated with antibiotics (0.4 μg/mL moenomycin, 5 μg/mL daptomycin, 2 μg/mL oxacillin, 2 μg/mL cefoxitin, or 2 μg/mL cefaclor) for 10 min at 37°C. Cell biomass was normalized based on OD_600_ of treated cultures, and a normalized biomass of treated cells was harvested immediately by centrifugation. Cell pellets were resuspended in 300 μL 1× PBS (pH 7.5) and 1.5 mL of 2:1 methanol:chloroform. The resuspended mixture was vortexed repetitively for 10 min at room temperature. Debris was pelleted by centrifugation (4,000 *g*, 10 min, room temperature). The supernatant was transferred to a new glass tube and mixed with 500 μL CHCl_3_ and 500 μL 1× PBS. The mixture was again vortexed for 10 min at room temperature, then centrifuged (4,000 *g*, 10 min, room temperature) to separate the organic and aqueous layers. The organic layer was collected, dried *in vacuo*, and resuspended in 20 μL dimethyl sulfoxide (DMSO).

Lipid II in the cellular lipid extract was labeled with biotin-d-lys (BDL) using penicillin-binding protein 4 (PBP4) as described (38, 42). A total of 2 μL of the lipid extracts was mixed with *S. aureus* PBP4 (10 μM final concentration) and BDL (4 mM final concentration) in reaction buffer (12.5 mM HEPES, 20 mM MnCl_2_, 2.5 mM Tween-80, pH 7.5) in a total volume of 10 μL. Mixtures were incubated for 1 h at room temperature. Reactions were stopped by adding 10 μL of 2× SDS loading buffer and were then resolved on a 4-20% TGX SDS-PAGE gel. The gel was transferred to an immunoblot PVDF membrane using a Transblot Turbo System with the preset Mixed MW setting. The membrane was blocked with Superblock T20 (TBS) for 1 h at room temperature. A 1:10,000 (vol/vol) dilution of Pierce streptavidin-HRP was added directly to the blocking buffer and equilibrated at room temperature for 1 h. The membrane was washed three times with wash buffer (1× TBS, 0.1% vol/vol Tween 20, pH 7.5) for 10 min each wash. Labeled lipid II was developed using Amersham ECL Prime Western Blotting Detection Reagent and visualized with BioMax Light Film.

### Reproducibility

Cysteine accessibility experiments were replicated three times in independent experiments. All co-IPs were replicated in at least two independent experiments, except for Fig. S1C, which was used to screen for a GpsB-binding motif and was independently validated by the results in Fig. 1E. To quantify the increase in PknB pulled down by the AuxB^W86A^ variant compared with wild type, we used two methods: densitometry of the Western blot and peptide counts of PknB in the total eluate fractions. The former gave a ratio of 4.2, and the latter gave a ratio of 5. The cysteine cross-linking experiment was replicated twice in independent experiments. Protein purifications were replicated at least twice in independent experiments. All spot titers were replicated in at least two independent experiments. The lipid II extraction and blot was performed once for β-lactams, but several different β-lactams were used and gave a consistent result. The lipid II extraction and blot was replicated twice for daptomycin. Myc-PknB and PknB-FLAG blots were each performed with two replicates. Each (phospho)protein data set was collected once with four biological replicates for each strain and condition. Each growth curve was measured once with three biological replicates for each strain and condition.

## SUPPLEMENTAL MATERIAL

Supplemental material mbio.01858-25-s0001.pdf Fig. S1-S5, captions for Tables S1-S5, plasmid and strain construction methods, and descriptions of strains used in this study.

Tables S1 and S2 mbio.01858-25-s0002.xlsx LC/MS data identifying PknB and GpsB as AuxB-interacting proteins.

Table S3 mbio.01858-25-s0003.xlsx LC/MS peptide counts identifying non-specific bands pulled down by AuxB.

Table S4 mbio.01858-25-s0004.xlsx Proteomics and phosphoproteomics data from PknB overexpression.

Table S5 mbio.01858-25-s0005.xlsx Proteomics and phosphoproteomics data from strains overexpressing inactive or truncated variants of PknB.

ASM does not own the copyrights to Supplemental Material that may be linked to, or accessed through, an article. The authors have granted ASM a non-exclusive, world-wide license to publish the Supplemental Material files. Please contact the corresponding author directly for reuse.
