# Supplementary material for: AuxB interacts directly with GpsB and PknB to coordinate cell envelope processes that contribute to intrinsic antibiotic resistance in Staphylococcus aureus
Source: mBio. 2025 Aug 25;16(10):e01858-25. doi: 10.1128/mbio.01858-25 (PMC12505970; doi:10.1128/mbio.01858-25)
Supplement: Supplemental material — Fig. S1-S5, captions for Tables S1-S5, plasmid and strain construction methods, and descriptions of strains used in this study. [file mbio.01858-25-s0001.pdf]

Supplemental Material For:

AuxB interacts directly with GpsB and PknB to coordinate cell envelope processes that contribute to intrinsic antibiotic resistance in *Staphylococcus aureus*

Tyler A. Sisley[1], Youngseon Park[1,‡], Ace George Santiago[1,‡], Wanassa Beroual[1], Isabella A. Sobolewski[1], Wonsik Lee[1,2], Joao A. Paulo[3], Suzanne Walker[1,*]

[1]Department of Microbiology, Blavatnik Institute, Harvard Medical School, Boston, Massachusetts 02115, USA.
[2]School of Pharmacy, Sungkyunkwan University, Suwon 16419, Republic of Korea
[3]Department of Cell Biology, Harvard Medical School, Boston, Massachusetts 02115, United States
[‡]These authors contributed equally
[*]To whom correspondence should be addressed (suzanne_walker@hms.harvard.edu)

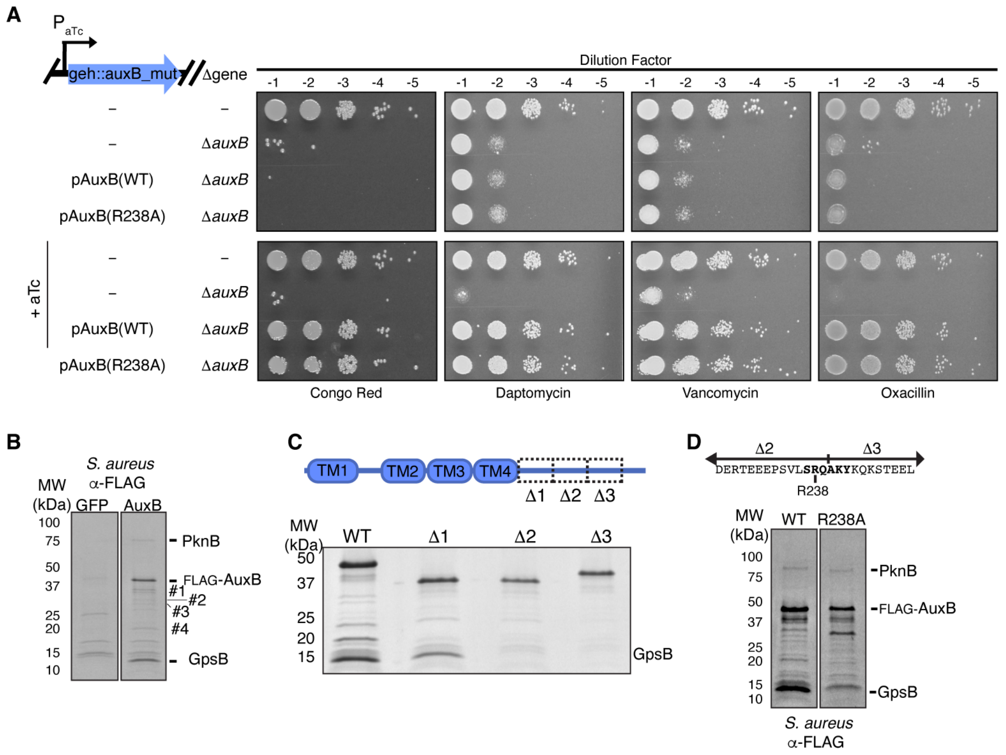

Figure S1. GpsB and PknB bind independently to AuxB.

(A) Spot assays of indicated Δ*auxB* complementation strains plated on various drugs with and without inducer. $AuxB^{WT}$ and $AuxB^{R238A}$, which does not bind GpsB, both complement on all Δ*auxB* drug conditions.

(B) Full gel image from Fig. 1D. AuxB, PknB, and GpsB are indicated. Marked bands were also excised and identified by LC/MS as the following proteins: #1 = AuxB degradation products; #2 = RplB; #3 = RplC; #4 = mixture of RplE, RplD, and Asp23 (Table S3). AlphaFold generated a low-confidence prediction of AuxB with Asp23, so it was not studied further.

(C) Coomassie stained gel of co-immunoprecipitated FLAG-AuxB variants. Membrane fractions were collected from *S. aureus* cells expressing FLAG-AuxB full length or FLAG-AuxB with the indicated internal deletions. Membranes were solubilized and isolated via FLAG magnetic beads. Δ1 corresponds to $AuxB^{\Delta Y160\text{-}N199}$, Δ2 corresponds to $AuxB^{\Delta T200\text{-}Q239}$, and Δ3 corresponds to $AuxB^{\Delta A240\text{-}I279}$.

(D) Full image of Coomassie stained gel sectioned in Fig. 1E. The $AuxB^{R238A}$ mutant, which cannot pull down GpsB, still pulls down PknB.

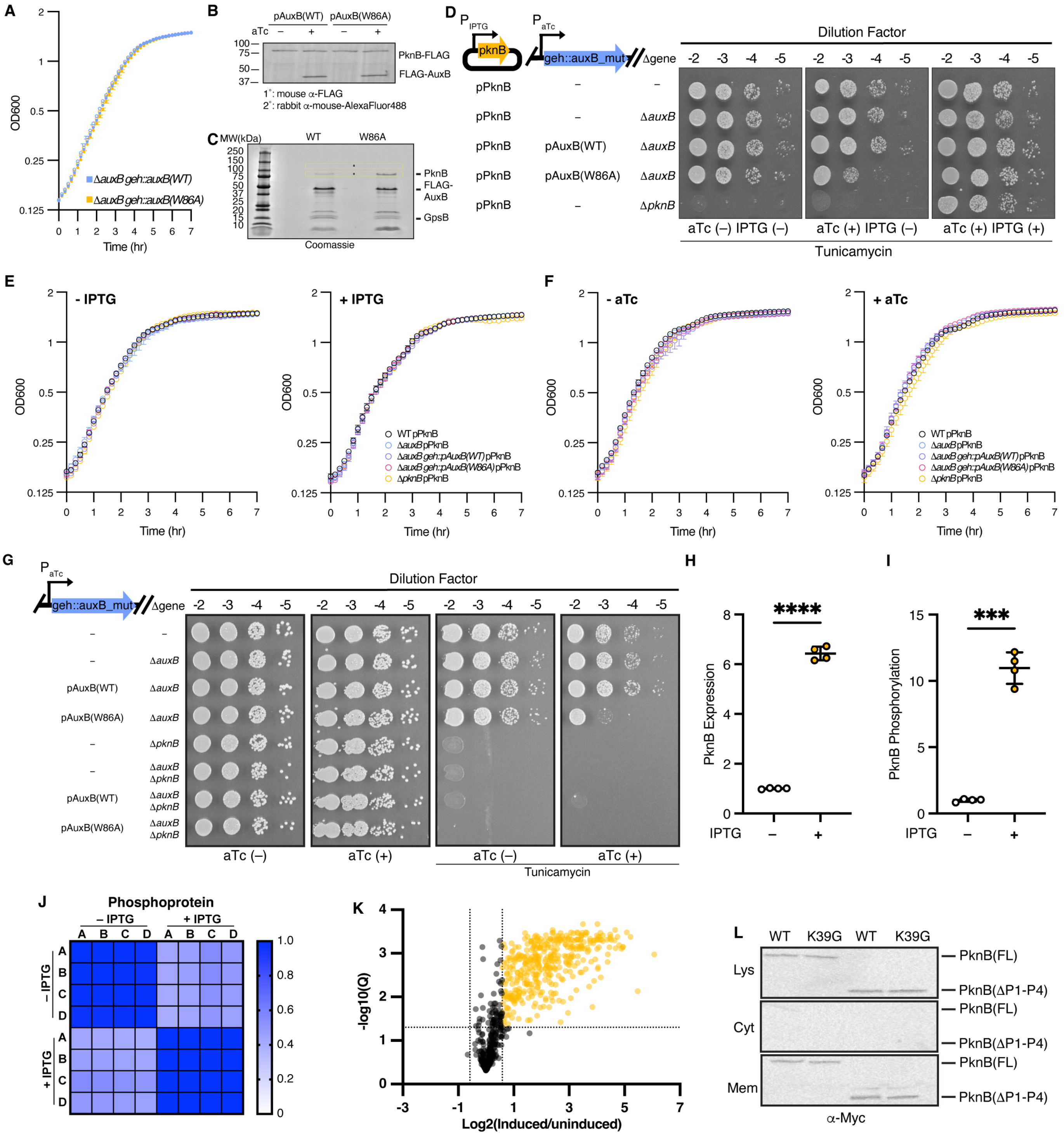

Figure S2. PknB is functional and properly localized to the membrane when overexpressed in *S. aureus*.

(A) Growth curve of Δ*auxB* complementation strains expressing AuxB^{WT} or AuxB^{W86A}. Exponentially growing strains were normalized to OD$_{600}$ = 0.1 and their growth was monitored. n = 3 biological replicates; points represent mean ± 1 standard deviation. Unfilled = uninduced. Filled = induced with 0.4 μM aTc.

(B) Western blot of PknB-FLAG and FLAG-AuxB in Δ*auxB* complementation strains expressing $AuxB^{WT}$ or $AuxB^{W86A}$. Exponentially growing strains were collected and assessed via α-FLAG immunoblot.

(C) Full image of the gel from Figure 2D. Yellow boxes show where densitometry was performed to compare the amounts of PknB pulled down. Background (top box) was subtracted from PknB bands (bottom box) to acquire values for comparison.

(D) Full image of tunicamycin experiment shown in Figure 3B. Tunicamycin = 0.4 μg/mL.

(E-F) Growth curves of strains from Figs. 3B and S2D. Strains were sub-cultured ± IPTG (E) or aTc (F). Exponentially growing strains were normalized to OD_{600} = 0.1 and their growth was monitored. n = 3 biological replicates; points represent mean ± 1 standard deviation.

(G) Spot assays of the indicated strains plated on tunicamycin (0.2 μg/mL). $AuxB^{W86A}$ is epistatic to Δ*pknB*, and the $AuxB^{W86A}$-induced sensitivity to tunicamycin is masked in a Δ*pknB* background.

(H-K) Strains harboring a PknB overexpression plasmid were cultured with or without inducer. Cultures were collected and processed for (phospho)proteomic analysis. n = 4 biological replicates.

(H) TMT values of all peptides mapping to PknB were quantified and summed.

(I) TMT values were quantified and summed for peptides with phosphorylation at T166.

(H-I) TMT signal was normalized to the average value of the uninduced control. Individual values are plotted with mean ± 1 standard deviation. Significance was determined using a paired t-test. ✱✱✱✱ = p<0.001, ✱✱✱ = p<0.005.

(J) Replicate consistency was determined by a Pearson correlation matrix. Phosphorylation is consistent between replicates of a given condition but not between conditions.

(K) Volcano plot of phosphosites identified in phosphoproteomics experiment. The plot is asymmetric, consistent with accumulation of phosphorylated substrates upon PknB overexpression. Significance was determined using a paired t-test. Phosphorylated proteins are listed in Table S4.

(L) Variants of PknB were expressed in Δ*pknB*. Cells were lysed and separated into cytoplasmic and membrane fractions. Fractions were resolved by gel electrophoresis and analyzed by Western Blot to compare protein abundance of PknB variants and ensure membrane localization. Lys = whole lysate fraction; Cyt = cytoplasmic fraction; Mem = membrane fraction. PknB(FL) = full-length PknB.

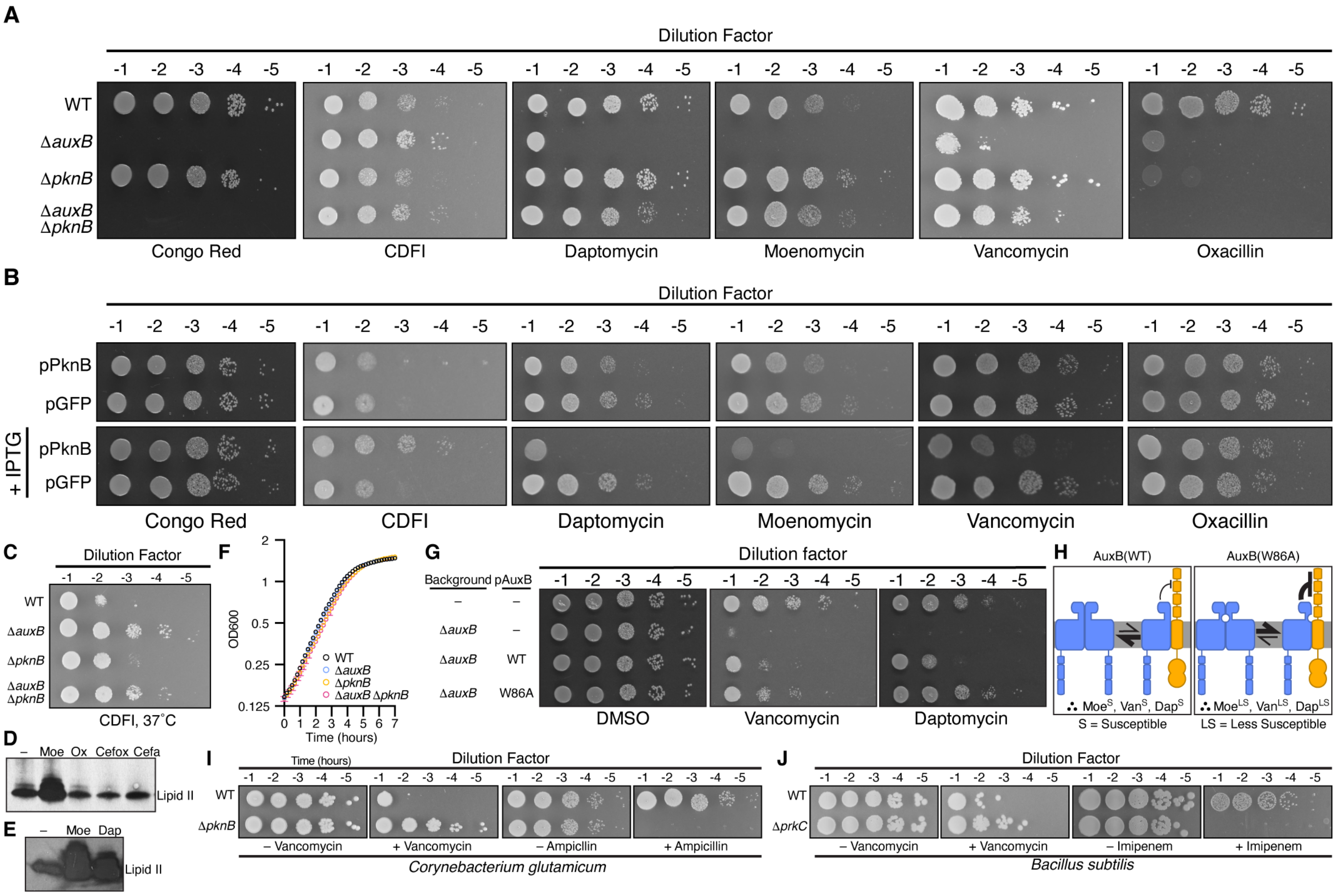

Figure S3. PknB activity increases susceptibility to drugs that accumulate Lipid II in the outer leaflet of the membrane.

(A-B) Full inhibitor panel tested in Fig. 3C-D. CDFI, moenomycin, and oxacillin plates are duplicate images from Fig. 3C-D for reference.

(A) Deletion of *pknB* suppresses Δ*auxB* sensitivity only on antibiotics that accumulate Lipid II in the outer leaflet of the membrane. Congo Red, 50 μg/mL; CDFI, 400 ng/mL; Daptomycin, 1.75 μg/mL; Moenomycin, 20 ng/mL; Vancomycin, 0.625 μg/mL; Oxacillin, 125 ng/mL.

(B) Strains overexpressing PknB or GFP were plated on the same compounds as in (A). Congo Red, 50 μg/mL; CDFI, 400 ng/mL; Daptomycin, 1.75 μg/mL; Moenomycin, 15 ng/mL; Vancomycin, 0.6 μg/mL; Oxacillin, 125 ng/mL.

(C) Deletion of *auxB* protects cells when translocation of Lipid II to the outer leaflet is inhibited. CDFI, 500 ng/mL at 37˚C.

(D-E) Exponentially growing *S. aureus* cultures were treated with compounds for 10 minutes. Lipid II was extracted and enzymatically labeled with biotin-D-lysine using *S. aureus* PBP4. Lipid II levels were assessed using a streptavidin HRP blot.

(D) "–" = untreated control; Moe = moenomycin; Ox = oxacillin; Cefox = cefoxatime; Cefa = cefaclor.

(E) "–" = untreated control; Moe = moenomycin; Dap = daptomycin.

(F) Growth curve of strains from Fig. 3C-D and S3A-C. Exponentially growing strains were normalized to OD_{600} = 0.1 and their growth was monitored. n = 3 biological replicates; points represent mean ± 1 standard deviation.

(G) Δ*auxB* complemented at an ectopic locus with either *auxB*^{WT} or *auxB*^{W86A} was plated on inducer and drug. Vancomycin, 0.75 μg/mL; Daptomycin, 1.75 μg/mL.

(H) Model for AuxB effect on drug susceptibility. Ectopic AuxB(WT) shows lower resistance to compounds that cause Lipid II to accumulate on the outer leaflet of the membrane (left). AuxB(W86A) binds and antagonizes more PknB, and this antagonism evidently protects cells against compounds that cause Lipid II to accumulate on the outer leaflet of the membrane (right).

(I) *Corynebacterium glutamicum* cells were plated on BHI agar without drug, with vancomycin (0.3 μg/mL) or with ampicillin (0.3 μg/mL).

(J) Exponentially growing *Bacillus subtilis* cells were plated on LB agar without drug, with vancomycin (0.16 μg/mL) or with imipenem (0.4 μg/mL)

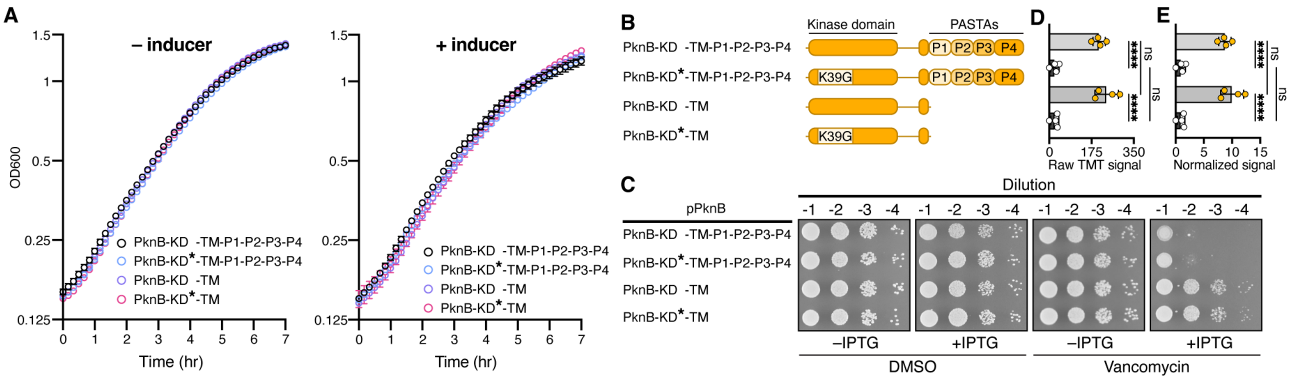

Figure S4. PknB's PASTA domain is toxic on Lipid II accumulating drugs and is not required for kinase activation.

(A) Growth curve of strains from Fig. 4C-E and S4C-E. Strains were sub-cultured ± IPTG. Exponentially growing strains were normalized to OD_{600} = 0.1 and their growth was monitored. n = 3 biological replicates; points represent mean ± 1 standard deviation.

(B) Reproduction of Fig. 4B. Linear diagram of PknB variants tested in Fig. S4C-E. KD* = Lys39Gly.

(C) Δ*pknB* strains containing a multi-copy plasmid that encodes one of the indicated PknB variants were plated on vancomycin (0.5 μg/mL).

(D-E) Plot of phosphorylation signal at $PknB^{T166}$ from the experiment described in Fig. 4D. n=4 biological replicates. Significance values determined by one-way ANOVA and Tukey's post-hoc analysis; ******** = p<0.001. All phosphorylated proteins are listed in Table S5.

(D) TMT signals corresponding to peptides with phosphorylated Thr166 were summed. Raw values are plotted with mean ± 1 standard deviation.

(E) Reproduction of Fig. 4D. Raw TMT values of $PknB^{WT}$ and $PknB^{K39G}$ were normalized to the average TMT signal of $PknB^{K39G}$. Raw TMT values of $PknB^{\Delta P1\text{-}P4}$ and $PknB^{K39G,\Delta P1\text{-}P4}$ were normalized to the average TMT signal of $PknB^{K39G,\Delta P1\text{-}P4}$. Normalized values are plotted with mean ± 1 standard deviation.

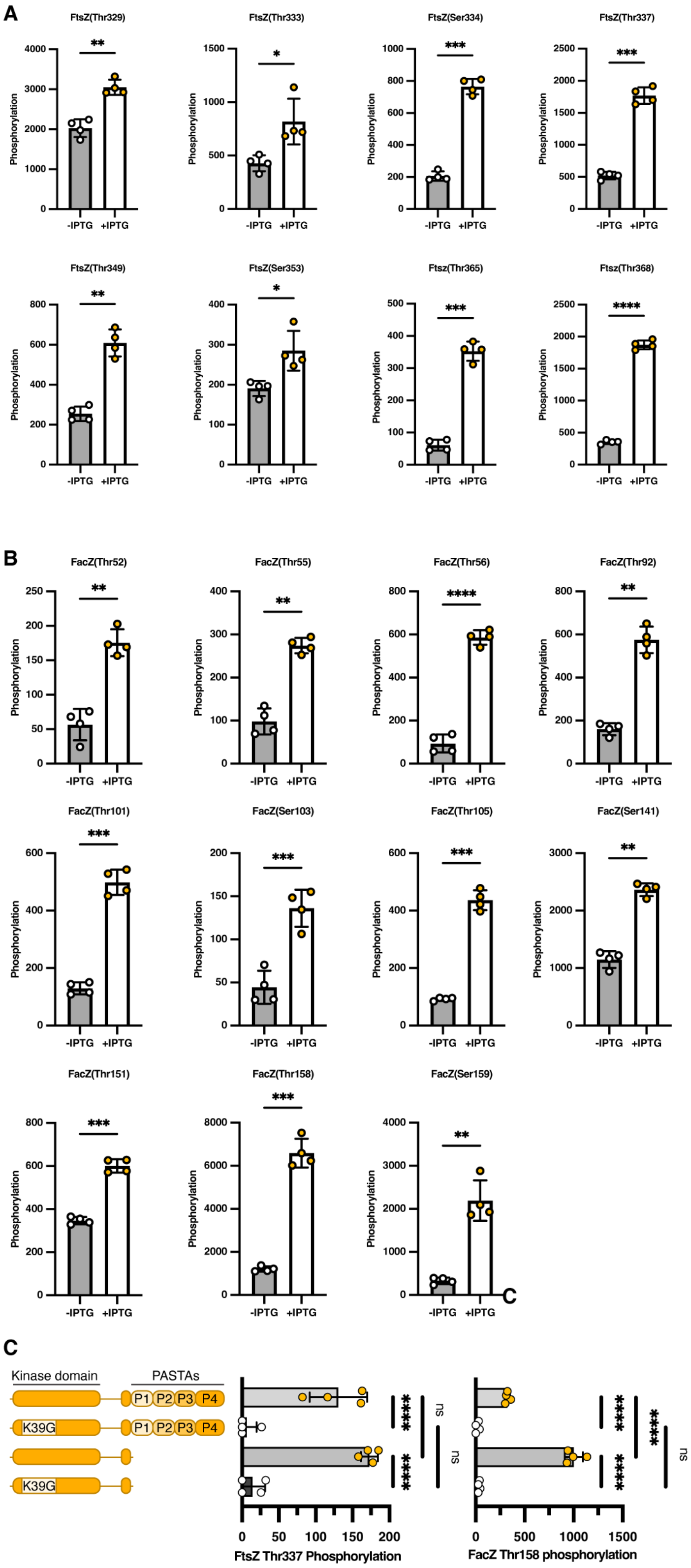

Figure S5. PknB phosphorylates the GpsB-binding proteins FtsZ and FacZ.

(A-B) Cultures were grown in the presence or absence of IPTG to induce PknB overexpression, and their phosphorylation profiles were assessed via quantitative phosphoproteomics. Phosphorylated peptides that mapped to FtsZ (A) and FacZ (B) were quantified and plotted. Significance was determined by a paired t-test. n = 4 biological replicates. Individual values are plotted with mean ± 1 standard deviation. ✱ = p<0.05. ✱✱ = p<0.01. ✱✱✱ = p<0.005. ✱✱✱✱ = p<0.001. Data presented are from the same experiment described in Fig. S2H-K.

(C) The indicated variants of PknB were expressed in a Δ*pknB* background. Phosphorylation profiles were determined by quantitative phosphoproteomics. Phosphorylated peptides that mapped to FtsZ (left) and FacZ (right) were quantified and plotted. n = 4 biological replicates. Individual values are plotted with mean ± 1 standard deviation. Significance was determined by a one-way ANOVA and Tukey's post-hoc analysis. ✱✱✱✱ = p<0.001. Data presented are from the same experiment described in Fig. 4D-E and Fig. S4D-E.

Table S1: Peptide counts identifying the 75 kDa band from Fig. 1D as PknB (SAOUHSC_01187). Eluates from a FLAG-AuxB pulldown were resolved by gel electrophoresis. The 75 kDa band was excised and identified by LC/MS.

Table S2: Peptide counts identifying the 15 kDa band from Fig. 1D as GpsB (SAOUHSC_01462). Eluates from a FLAG-AuxB pulldown were resolved by gel electrophoresis. The 15 kDa band was excised and identified by LC/MS.

Table S3: Peptide counts identifying intermediate molecular weight bands from Fig. S1B as contaminants. Various bands and sections were excised and identified by LC/MS.

Table S4: Quantitative proteomic and phosphoproteomic data from PknB-overexpressing strains. S. aureus harboring a multicopy plasmid carrying wildtype *pknB* were grown with and without inducer. Cells were lysed in the presence of phosphatase inhibitors, and proteins were collected and assessed by TMT-labeling and LC/MS analysis. Significance values for each protein or phosphosite were determined using a paired t-test.

Table S5: Quantitative proteomic and phosphoproteomic data from PknB variant-overexpressing strains. S. aureus harboring a multicopy plasmid carrying wildtype, inactive, and truncated variants of *pknB* were grown with inducer. Cells were lysed in the presence of phosphatase inhibitors, and proteins were collected and assessed by TMT-labeling and LC/MS analysis. Significance values for each protein or phosphosite were determined using a one-way ANOVA with a post-hoc Tukey's analysis.

SUPPLEMENTARY METHOTHODS

S. aureus Strain construction:

The Δ*pknB* and *pknB-FLAG* strains from this study was generated in RN4220 via a one-step deletion method (1). A resistance cassette with short homology arms was amplified from an unpublished plasmid (pJP60) using oTS249 and oTS250 for Δ*pknB*. For *pknB-FLAG*, the pJP60 resistance cassette was amplified first by oTS254 and oTS435 to install short homology arms and then amplified again by oTS436 and oTS437 to extend the homology arms. 1 μg clean PCR product was transformed into RN4220. Selection was performed on TSA supplemented with 50 μg/mL kanamycin and 50 μg/mL neomycin and grown at 37˚C for 1-2 days until colonies formed.

pLOW plasmids were introduced into *S. aureus* by electroporation into competent RN4220 using a BioRad GenePulser Xcell (settings: 2900 V, 25 μF, 100Ω). Immediately after electroporation, 1 mL of TSB was added directly to the electroporation cuvette. Samples were transferred to a 1.5 mL Eppendorf tube and incubated at 37˚C for 1.5-2 hr to allow outgrowth. This culture was plated onto TSA supplemented with 10 μg/mL erythromycin and grown at 37˚C for 1-2 days until colonies formed.

pTP63 plasmids were introduced into *S. aureus* by electroporation into competent RN4220 containing pTP44 (2) using a BioRad GenePulser Xcell (settings: 2900 V, 25 μF, 100Ω). Immediately after electroporation, 1 mL of TSB was added directly to the electroporation cuvette. Samples were transferred to a 1.5 mL Eppendorf tube and incubated at 37˚C for 1.5-2 hr to allow outgrowth. This culture was plated onto TSA supplemented with 10 μg/mL chloramphenicol and grown at 37˚C for 1 day until colonies formed.

To generate strains in *S. aureus* HG003, we first generated ϕ85 lysates from donor *S. aureus* RN4220 strains. Overnight cultures of donor bacteria were used to generate lysates. Overnight cultures were sub-cultured 1:100 (v/v) in fresh TSB and grown to OD_{600} = 0.2-0.5 and were normalized to OD_{600} = 0.2. 300 μL donor culture was mixed with 5 μL 1M $CaCl_2$ and 200 μL ϕ85 phage (dilution of phage varies depending on titer required to produce a lacy pattern). The phage and bacteria were incubated together at room temperature for 30 min before 6 mL of melted "top agar" (TSB with 0.75% w/v agar supplemented with 7 mM $CaCl_2$) was added directly to the sample. The sample and top agar were mixed briefly and then poured atop "bottom agar" (TSB with 1.5% w/v agar supplemented with 10 mM $CaCl_2$). Once the top agar solidified, the plate was incubated at 30˚C overnight until a lacy pattern of bacterial growth was present. To harvest ϕ85 lysates, 10 mL TSB was added to the plate, which was rocked side-to-side for 16-24 hr at room temperature. ϕ85 lysates were collected, debris was pelleted by centrifugation (12,000*g* for 10 min), and the supernatant was filter-sterilized using a 0.2 μm pore PVDF sterile filter (Genesee).

Donor ϕ85 lysates were then used to transduce plasmids or deletions into recipient *S. aureus* HG003 strains. Overnight cultures of recipient strains were concentrated 20x in TSB. 50 μL of the concentrated recipient strain was mixed with 2 μL 1 M $CaCl_2$, 100 μL of TSB, and 50 μL of donor ϕ85 lysate, then mixed briefly. Samples were incubated at 37˚C for 25 min without shaking and an additional 15 min while shaking at 300 rpm. The transduction was stopped by adding 500 μL of ice cold 0.02 M sodium citrate. Cells were pelleted by centrifugation (10,000*g*, 2 min) and supernatant was removed. Pellets were resuspended in 100 μL ice cold 0.02 M sodium citrate and incubated for 1-2 hr on ice before being plated on TSA supplemented with 0.05% (w/v) sodium citrate and appropriate antibiotics (10 μg/mL erythromycin for selection of pLOW; 10 μg/mL chloramphenicol for selection of pTP63; 50 μg/mL kanamycin and 50 μg/mL neomycin for Δ*pknB*). Single colonies were cured of phage by re-streaking on TSA supplemented with 0.05% (w/v) sodium citrate and appropriate antibiotics.

***C. glutamicum* Strain construction:**

The Δ*pknB* strain from this study was generated in MB001 via a one-step deletion method (3). The resistance cassette used was amplified from pEWL74 using oWB48 and oWB49. Selection was performed on BHI supplemented with 9.1% (w/v) sorbitol and 15 μg/mL kanamycin and grown at 30˚C until colonies formed.

Plasmid Construction

pAGS101: This plasmid was used for IPTG-inducible expression of C-terminally FLAG-tagged GFP for compound sensitivity studies in *S. aureus*. DNA encoding GFPmut2 was amplified (oligos AGS-11 and AGS-12) from an unpublished plasmid (pAGS100) by PCR. Vector DNA was obtained from empty pLOW. PCR and vector DNA were digested with BamHI and SalI. Digested pieces were joined by T4 ligation.

pAGS102 (This plasmid was not used directly but was used in the construction of pAGS103): This plasmid is capable of heterologous overexpression and purification of N-terminally His-tagged PknB in *E. coli*. Empty pETDuet1 was used as the source of backbone. DNA encoding PknB was amplified (oligos AGS-17 and AGS-18) from *S. aureus* genomic DNA by PCR. PCR and vector DNA were digested with BamHI and SalI. Digested pieces were joined by T4 ligation.

pAGS103: This plasmid was used for heterologous overexpression and co-purification of N-terminally His-tagged PknB and N-terminally FLAG-tagged AuxB in *E. coli*. pAGS102 was used as the source of

backbone. DNA encoding AuxB was amplified (oligos AGS-19 and AGS-21) from *S. aureus* genomic DNA by PCR. PCR and vector DNA were digested with NdeI and KpnI. Digested pieces were joined by T4 ligation.

pAGS104: This plasmid was used for heterologous overexpression and purification of N-terminally FLAG-tagged AuxB in *E. coli*. Empty pETDuet1 was used as the source of backbone. DNA encoding AuxB was amplified (oligos AGS-19 and AGS-21) from *S. aureus* genomic DNA by PCR. PCR and vector DNA were digested with NdeI and KpnI. Digested pieces were joined by T4 ligation.

pAGS105 (This plasmid was not used directly but was used in the construction of pAGS106): AuxB was amplified (oligos AGS-114 and AGS-115) from *S. aureus* genomic DNA by PCR. The vector was obtained from empty pLOW. The PCR and the vector DNA were digested with SalI and EcoRI. Digested pieces were joined by T4 ligation.

pAGS106 (This plasmid was not used directly but was used in the construction of pTS052 and pTS054): pAGS105 was amplified in two fragments (Fragment 1 by oligos AGS-117 and AGS-121; Fragment 2 by oligos AGS-119 and AGS-120) by PCR. PCRs resulted in overlapping fragments that were joined by a single step In-Fusion assembly.

pAGS107: This plasmid was used for aTc-inducible expression of C-terminally FLAG-tagged GFP for use in pulldown studies. GFP was amplified from pTD31 (4) (oligos AGS-2 and AGS-3) by PCR. Vector was obtained from empty pTP63. The PCR and the vector DNA were digested with KpnI and EcoRI. Digested pieces were joined by T4 ligation.

pTS040: This plasmids were used for aTc-inducible expression of N-terminally FLAG-tagged AuxB for complementation studies and pulldowns in *S. aureus*. Full length AuxB was amplified from an unpublished plasmid (pTS014, oligos oTS091 and oTS093) by PCR. This PCR product was used as template to amplify full length AuxB (oligos oTS092 and oTS093). The vector was derived from an unpublished plasmid (pTS006). The PCR product and vector were digested with BlpI and KpnI. The digested PCR product and vector were joined together by T4 ligation.

pTS052 (used to construct pTS053): The pAGS106 (pLOW) backbone was amplified (oligos oTS128 and oTS134) by PCR. AuxB was amplified from an unpublished plasmid (pTS014, oligos oTS129 and

oTS137) by PCR. PCRs resulted in overlapping regions between DNA fragments and were assembled via a one step In-Fusion assembly.

pTS053: This plasmid was used for IPTG-inducible expression of an N-terminally FLAG-tagged AuxB variant lacking all native cysteine residues for SCAM experiments in *S. aureus*. The backbone (oligos oTS113 and oTS109) and insert (oligos oTS114 and oTS115) were amplified from pTS052 by PCR. PCRs resulted in overlapping regions between the DNA fragments and were joined via one step In-Fusion assembly.

pTS054: This plasmid was used for IPTG-inducible expression of an N-terminally FLAG-tagged AuxB variant lacking all native cysteine residues with non-native cysteine substitution for SCAM experiments in *S. aureus*. The backbone (oligos oTS113 and oTS109) and insert (oligos oTS114 and oTS115) were amplified from pAGS106 by PCR. PCRs resulted in overlapping regions between the DNA fragments and were assembled via a one step In-Fusion assembly.

pTS055 and pTS056: These plasmids were used for IPTG-inducible expression of N-terminally FLAG-tagged AuxB variants lacking all native cysteine residues with non-native cysteine substitutions for SCAM experiments in *S. aureus*. The entirety of the plasmid (oligos oTS125 and oTS135 for pTS055; oligos oTS127 and oTS136 for pTS056) was amplified from pTS053. PCRs resulted in non-homologous ends that were joined by blunt end ligation.

pTS063-pTS065: These plasmids were used for aTc-inducible expression of N-terminally FLAG-tagged AuxB internal deletion variants for pulldown experiments in *S. aureus*. pTS040 in its entirety was amplified (oligos oTS163 and oTS164 for pTS063; oligos oTS165 and oTS166 for pTS064; oligos oTS167 and oTS168 for pTS065) by PCR. PCRs resulted in non-homologous ends that were joined by blunt end ligation.

pTS070: This plasmid was used for aTc inducible expression of an N-terminally FLAG-tagged AuxB single-amino acid substitution for complementation studies and pulldowns in *S. aureus*. pTS040 was amplified (oligos oTS207 and oTS208) in its entirety by PCR. The PCR resulted in homologous ends that were joined by a single step In-Fusion reaction.

pTS075: This plasmid was used for aTc inducible expression of an N-terminally FLAG-tagged AuxB single-amino acid substitution for complementation studies and pulldown experiments in *S. aureus*. pTS040 was amplified (oligos oTS219 and oTS220) in its entirety by PCR. The PCR resulted in homologous ends that were joined by a single step In-Fusion reaction.

pTS090: This plasmid was used for IPTG-inducible dual expression and co-purification of N-terminally His_6-tagged PknB and N-terminally FLAG-tagged $AuxB^{W86A}$. pAGS103 was amplified (oligos oTS219 and oTS220) in its entirety by PCR. The PCR resulted in homologous ends that were joined by a single step In-Fusion reaction.

pTS091: This plasmid was used for IPTG-inducible expression of N-terminally Myc-tagged PknB for complementation experiments and phenotypic profiling in *S. aureus*. pTS091 was generated by a four piece isothermal assembly. Fragment 1 (oligos oTS297 and AMo81), Fragment 2 (oligos AMo82 and AMo83), Fragment 3 (oligos AMo84 and oTS298) were amplified from pTS052 by PCR. DNA encoding PknB was amplified (oligos oTS295 and oTS296) from *S. aureus* genomic DNA by PCR. PCRs resulted in overlapping regions of DNA that were joined by a one-step In-Fusion assembly.

pTS123: This plasmid was used for aTc-inducible expression of an N-terminally FLAG-tagged AuxB variant lacking all native cysteine residues to determine dimerization state of AuxB in *S. aureus*. The backbone (oligos oTS113 and oTS109) and insert (oligos oTS114 and oTS115) were amplified from pTS040 by PCR. PCRs resulted in overlapping regions between the DNA fragments and were joined via one step In-Fusion assembly.

pTS124-pTS127: These plasmids were used for aTc-inducible expression of N-terminally FLAG-tagged AuxB variants lacking all native cysteine residues and with single-amino acid cysteine substitutions to determine dimerization state of AuxB in *S. aureus*. The entirety of pTS123 was amplified (oligos oTS373 and oTS374 for pTS124; oligos oTS359 and oTS360 for pTS125; oligos oTS361 and oTS362 for pTS126; oligos oTS363 and oTS364 for pTS127) by PCR. The PCRs resulted in homologous ends that were joined by a single step In-Fusion assembly.

pTS163: This plasmid was used for IPTG-inducible expression of the N-terminally Myc-tagged PknB variant lacking its PASTA domain. pTS091 was amplified (oligos oTS297 and oTS608) in its entirety by PCR. The PCRs resulted in homologous ends that were joined by a single step In-Fusion reaction.

pTS182: This plasmid was used for IPTG-inducible expression of the full length PknB variant with an N-terminal Myc-tag and a Lys39Gly amino acid substitution in the kinase domain. pTS091 was amplified (oligos oTS631 and oTS632) in its entirety by PCR. The PCRs resulted in homologous ends that were joined by a single step In-Fusion reaction.

pTS203: This plasmid was used for IPTG-inducible expression of the N-terminally Myc-tagged PknB variant lacking its PASTA domain and with the Lys39Gly amino acid substitution in the kinase domain. pTS163 was amplified (oligos oTS631 and oTS632) in its entirety by PCR. The PCRs resulted in homologous ends that were joined by a single step In-Fusion reaction.

Bacterial Strains, Plasmids and Primers

***S. aureus* Strain**	**Background**	**Genotype**	**Source**	**Construction Notes**
AGS030	HG003	Δ*auxB::tet*	Coe, K *et al.*(5)	
aTP394	HG003	spa::Tn-erm	Pang, T *et al.*(2)	
TAS006	HG003	WT		
TAS007	RN4220	WT		
TAS008	RN4220	pTP44	Pang, T *et al.*(2)	pTP44 contains the integrase required for pTP63 integration into the L54a *attB* site in the *S. aureus geh* locus.
AGS040	HG003	Δ*auxB::tet* geh::pTP63-rbs$_{auxB}$-GFP-FLAG spa::Tn-erm	This Study	Φ85 lysate generated from AGS030 was used to transduce into aTP394[2] to generate HG003 Δ*auxB::tet* spa::Tn-erm. pTP63-rbs$_{auxB}$-GFP-FLAG was transformed into TAS008. Φ85 lysate generated from this strain was transduced into the intermediate strain to generate AGS040.
AGS042	HG003	Δ*auxB::tet* geh::pTP63-rbs$_{auxB}$-FLAG-AuxB^{WT} spa::Tn-erm	This Study	Φ85 lysate generated from AGS030 was used to transduce into aTP394[2] to generate HG003 Δ*auxB::tet* spa::Tn-erm. Φ85 lysate generated from an unpublished strain (ARH127) encoding pTP63-rbs$_{auxB}$-FLAG-AuxB^{WT} was used to transduced into the intermediate strain to generate AGS042.
TAS122	HG003	Δ*auxB::tet* pLOW-rbs$_{rpoB}$-FLAG-auxB^{C25S,C95S,A304C}	This study	pLOW-rbs$_{rpoB}$-FLAG-auxB^{C25S,C95S,A304C} was transformed into TAS007. Φ85 lysate generated from this strain was used to transduce into AGS030 to generate TAS122.
TAS123	HG003	Δ*auxB::tet* pLOW-rbs$_{rpoB}$-FLAG-auxB^{C25S,C95S,S14C}	This study	pLOW-rbs$_{rpoB}$-FLAG-auxB^{C25S,C95S,S14C} was transformed into TAS007. Φ85 lysate generated from this strain was used to transduce into AGS030 to generate TAS123.
TAS124	HG003	Δ*auxB::tet* pLOW-rbs$_{rpoB}$-FLAG-auxB^{C25S,C95S,S49C}	This study	pLOW-rbs$_{rpoB}$-FLAG-auxB^{C25S,C95S,S49C} was transformed into TAS007. Φ85 lysate generated from this strain was used to transduce into AGS030 to generate TAS124.

TAS126	HG003	Δ*auxB::tet geh::*pTP63-rbs$_{auxB}$-FLAG-auxB^{WT}	This study	pTP63-rbs$_{auxB}$-FLAG-auxB^{WT} was transformed into TAS008. Φ85 lysate generated from this strain was used to transduce into AGS030 to generate TAS126.
TAS156	RN4220	geh::pTP63-rbs$_{auxB}$-FLAG-auxB$^{\Delta Y160\text{-}N199}$	This study	pTP63-rbs$_{auxB}$-FLAG-auxB $^{\Delta Y160\text{-}N199}$ was transformed into TAS008.
TAS157	RN4220	geh::pTP63-rbs$_{auxB}$-FLAG-auxB$^{\Delta T200\text{-}Q239}$	This study	pTP63-rbs$_{auxB}$-FLAG-auxB$^{\Delta T200\text{-}Q239}$ was transformed into TAS008.
TAS158	RN4220	geh::pTP63-rbs$_{auxB}$-FLAG-auxB$^{\Delta A240\text{-}I279}$	This study	pTP63-rbs$_{auxB}$-FLAG-auxB$^{\Delta A240\text{-}I279}$ was transformed into TAS008.
TAS172	HG003	Δ*auxB::tet* pLOW-rbs$_{rpoB}$-FLAG-auxB^{C25S,C95S}	This study	pLOW-rbs$_{rpoB}$-FLAG-auxB^{C25S,C95S} was transformed into TAS007. Φ85 lysate generated from this strain was used to transduce into AGS030 to generate TAS172.
TAS204	HG003	Δ*auxB::tet* geh::pTP63-rbs$_{auxB}$-FLAG-auxB^{R238A}	This study	pTP63-rbs$_{auxB}$-FLAG-auxB^{R238A} was transformed into TAS008. Φ85 lysate generated from this strain was used to transduce into AGS030 to generate TAS204.
TAS207	HG003	Δ*auxB::tet* geh::pTP63-rbs$_{auxB}$-FLAG-auxB^{W86A}	This study	pTP63-rbs$_{auxB}$-FLAG-auxB^{W86A} was transformed into TAS008. Φ85 lysate generated from this strain was used to transduce into AGS030 to generate TAS207.
TAS214	RN4220	RN4220 pTW07 Δ*pknB::Kan*	This study	Made by homologous recombination into RN4220.
TAS215	HG003	Δ*pknB::Kan*	This study	Φ85 lysate generated from TAS214 was used to transduce into TAS006.
TAS228	HG003	Δ*auxB::tet* Δ*pknB::Kan*	This study	Φ85 lysate generated from TAS214 was used to transduce into AGS030.
TAS245	HG003	pLOW-rbs$_{rpoB}$-Myc-PknB	This study	pLOW-rbs$_{rpoB}$-Myc-PknB was transformed into TAS007. Φ85 lysate generated from this strain was used to transduce into TAS006 to generate TAS245.
TAS246	HG003	Δ*auxB::tet* pLOW-rbs$_{rpoB}$-Myc-PknB	This study	pLOW-rbs$_{rpoB}$-Myc-PknB was transformed into TAS007. Φ85 lysate generated from this strain was used to transduce into AGS030 to generate TAS246.
TAS247	HG003	Δ*auxB::tet* geh::pTP63-rbs$_{auxB}$-FLAG-auxB^{WT} pLOW-rbs$_{rpoB}$-Myc-PknB	This study	pLOW-rbs$_{rpoB}$-Myc-PknB was transformed into TAS007. Φ85 lysate generated from this strain was used to transduce into TAS126 to generate TAS247.
TAS248	HG003	Δ*auxB::tet* geh::pTP63-rbs$_{auxB}$-FLAG-auxB^{W86A} pLOW-rbs$_{rpoB}$-Myc-PknB	This study	pLOW-rbs$_{rpoB}$-Myc-PknB was transformed into TAS007. Φ85 lysate generated from this strain was used to transduce into TAS207 to generate TAS248.
TAS249	HG003	Δ*pknB::Kan* pLOW-rbs$_{rpoB}$-Myc-PknB	This study	pLOW-rbs$_{rpoB}$-Myc-PknB was transformed into TAS007. Φ85 lysate generated from this strain was used to transduce into TAS215 to generate TAS249.
TAS250	HG003	pLOW-rbs$_{rpoB}$-GFP-FLAG	This study	pLOW-rbs$_{rpoB}$-GFP-FLAG was transformed into TAS007. Φ85 lysate generated from this strain was used to transduce into TAS006 to generate TAS250.
TAS268	HG003	Δ*auxB::tet* geh::pTP63-rbs$_{auxB}$-FLAG-auxB^{C25S,C95S}	This study	pTP63-FLAG-auxB^{C25S,C95S} was transformed into TAS008. Φ85 lysate generated from this strain was used to transduce into AGS030 to generate TAS268.
TAS269	HG003	Δ*auxB::tet* geh::pTP63-rbs$_{auxB}$-FLAG-auxB^{C25S,C95S,W86C}	This study	pTP63-FLAG-auxB^{C25S,C95S,W86C} was transformed into TAS008. Φ85 lysate generated from this strain was used to transduce into AGS030 to generate TAS269.
TAS270	HG003	Δ*auxB::tet* geh::pTP63-rbs$_{auxB}$-FLAG-auxB^{C25S,C95S,I93C}	This study	pTP63-FLAG-auxB^{C25S,C95S,I93C} was transformed into TAS008. Φ85 lysate generated from this strain was used to transduce into AGS030 to generate TAS270.
TAS271	HG003	Δ*auxB::tet* geh::pTP63-rbs$_{auxB}$-FLAG-auxB^{C25S,C95S,L97C}	This study	pTP63-FLAG-auxB^{C25S,C95S,L97C} was transformed into TAS008. Φ85 lysate generated from this strain was used to transduce into AGS030 to generate TAS271.

TAS272	HG003	Δ*auxB::tet* geh::pTP63-rbs$_{auxB}$-FLAG-auxB^{C25S,C95S,F100C}	This study	pTP63-FLAG-auxB^{C25S,C95S,F100C} was transformed into TAS008. Φ85 lysate generated from this strain was used to transduce into AGS030 to generate TAS272.
TAS326	RN4220	*pknB-FLAG::Kan*	This study	Made by homologous recombination into RN4220.
TAS330	HG003	Δ*auxB::tet pknB-FLAG::Kan*	This study	Φ85 lysate generated from TAS326 was used to transduce into AGS030 to generate TAS330.
TAS461	HG003	Δ*pknB::Kan* pLOW-rbs$_{rpoB}$-Myc-PknB^{K39G}	This study	pLOW-rbs$_{rpoB}$-Myc-PknB^{K39G} was transformed into TAS007. Φ85 lysate generated from this strain was used to transduce into TAS215 to generate TAS461.
TAS472	HG003	Δ*pknB::Kan* pLOW-rbs$_{rpoB}$-Myc-PknB$^{\Delta Y376\text{-}V664}$	This study	pLOW-rbs$_{rpoB}$-Myc- PknB$^{\Delta Y376\text{-}V664}$ was transformed into TAS007. Φ85 lysate generated from this strain was used to transduce into TAS215 to generate TAS472.
TAS477	HG003	Δ*pknB::Kan* pLOW-rbs$_{rpoB}$-Myc-PknB$^{K39G,\Delta Y376\text{-}V664}$	This study	pLOW-rbs$_{rpoB}$-Myc- PknB$^{K39G,\Delta Y376\text{-}V664}$ was transformed into TAS007. Φ85 lysate generated from this strain was used to transduce into TAS215 to generate TAS477.
TAS514	HG003	Δ*auxB::tet geh::*pTP63-rbs$_{auxB}$-FLAG-auxB^{WT} *pknB-FLAG::Kan*	This study	pTP63-rbs$_{auxB}$-FLAG-auxB^{WT} was transformed into TAS008. Φ85 lysate generated from this strain was used to transduce into TAS330 to generate TAS514.
TAS515	HG003	Δ*auxB::tet* geh::pTP63-rbs$_{auxB}$-FLAG-auxB^{W86A} *pknB-FLAG::Kan*	This study	pTP63-rbs$_{auxB}$-FLAG-auxB^{W86A} was transformed into TAS008. Φ85 lysate generated from this strain was used to transduce into TAS330 to generate TAS515.
TAS516	HG003	Δ*auxB::tet geh::*pTP63-rbs$_{auxB}$-FLAG-auxB^{WT} Δ*pknB::Kan*	This study	Φ85 lysate generated from TAS214 was used to transduce into TAS126 to make TAS516.
TAS517	HG003	Δ*auxB::tet* geh::pTP63-rbs$_{auxB}$-FLAG-auxB^{W86A} Δ*pknB::Kan*	This study	Φ85 lysate generated from TAS214 was used to transduce into TAS207 to make TAS517.

**C. glutamicum Strain**	**Background**	**Genotype**	**Source**	**Construction Notes**
H60	MB001	WT	Baumgart, M et al. (6)	Obtained from the Bernhardt Lab at Harvard Medical School.
H2813	MB001	Δ*pknB::Kan*	This study	Constructed through homologous recombination into MB001.

**B. subtilis Strain**	**Background**	**Genotype**	**Source**	**Construction Notes**
TAS302	Strain 168	WT		Obtained from the Rudner Lab at Harvard Medical School.
TAS306	Strain 168	Δ*prkC*	Koo, B et al. (7)	

**Plasmid**	**Genotype & Description**	***E. coli* Marker**	***S. aureus* Marker**	**Source**
pETDuet1	*amp^R* vector for dual expression in *E. coli*	Ampicillin		EMD Millipore
pAGS101	pLOW-rbs$_{rpoB}$-GFP-FLAG under a P$_{spac}$ promoter	Ampicillin	Erythromycin	This study
pAGS102	pETDuet1-[MCS1: His$_6$-PknB; MCS2: Empty]	Ampicillin		This study
pAGS103	pETDuet1-[MCS1: His$_6$-PknB; MCS2: FLAG-AuxB]	Ampicillin		This study
pAGS104	pETDuet1-[MCS1: Empty; MCS2: FLAG-AuxB]	Ampicillin		This study
pAGS105	pLOW-rbs$_{rpoB}$-FLAG-auxB under a P$_{spac}$ promoter	Ampicillin	Erythromycin	This study
pAGS106	pLOW-rbs$_{rpoB}$-FLAG-auxB^{A304C} under a P$_{spac}$ promoter	Ampicillin	Erythromycin	This study
pAGS107	pTP63-rbs$_{auxB}$-GFP-FLAG under a P$_{tet}$ promoter	Ampicillin	Chloramphenicol	This study
pTS040	pTP63-rbs$_{auxB}$-FLAG-auxB under a P$_{tet}$ promoter	Ampicillin	Chloramphenicol	This study

pTS052	pLOW-rbs$_{rpoB}$-FLAG-auxB under a P$_{spac}$ promoter	Ampicillin	Erythromycin	This study
pTS053	pLOW-rbs$_{rpoB}$-FLAG-auxB^{C25S,C95S} under a P$_{spac}$ promoter	Ampicillin	Erythromycin	This study
pTS054	pLOW-rbs$_{rpoB}$-FLAG-auxB^{C25S,C95S,A304C} under a P$_{spac}$ promoter	Ampicillin	Erythromycin	This study
pTS055	pLOW-rbs$_{rpoB}$-FLAG-auxB^{C25S,C95S,S14C} under a P$_{spac}$ promoter	Ampicillin	Erythromycin	This study
pTS056	pLOW-rbs$_{rpoB}$-FLAG-auxB^{C25S,C95S,S49C} under a P$_{spac}$ promoter	Ampicillin	Erythromycin	This study
pTS063	pTP63-rbs$_{auxB}$-FLAG-auxB$^{\Delta Y160\text{-}N199}$ under a P$_{tet}$ promoter	Ampicillin	Chloramphenicol	This study
pTS064	pTP63-rbs$_{auxB}$-FLAG-auxB$^{\Delta T200\text{-}Q239}$ under a P$_{tet}$ promoter	Ampicillin	Chloramphenicol	This study
pTS065	pTP63-rbs$_{auxB}$-FLAG-auxB$^{\Delta A240\text{-}I279}$ under a P$_{tet}$ promoter	Ampicillin	Chloramphenicol	This study
pTS070	pTP63-rbs$_{auxB}$-FLAG-auxB^{R238A} under a P$_{tet}$ promoter	Ampicillin	Chloramphenicol	This study
pTS075	pTP63-rbs$_{auxB}$-FLAG-auxB^{W86A} under a P$_{tet}$ promoter	Ampicillin	Chloramphenicol	This study
pTS090	pETDuet1-[MCS1: His$_6$-PknB; MCS2: FLAG-AuxB^{W86A}]	Ampicillin		This study
pTS091	pLOW-rbs$_{rpoB}$-Myc-PknB under a P$_{spac}$ promoter	Ampicillin	Erythromycin	This study
pTS123	pTP63-rbs$_{auxB}$-FLAG-auxB^{C25S,C95S} under a P$_{tet}$ promoter	Ampicillin	Chloramphenicol	This study
pTS124	pTP63-rbs$_{auxB}$-FLAG-auxB^{C25S,C95S,W86C} under a P$_{tet}$ promoter	Ampicillin	Chloramphenicol	This study
pTS125	pTP63-rbs$_{auxB}$-FLAG-auxB^{C25S,C95S,I93C} under a P$_{tet}$ promoter	Ampicillin	Chloramphenicol	This study
pTS126	pTP63-rbs$_{auxB}$-FLAG-auxB^{C25S,C95S,L97C} under a P$_{tet}$ promoter	Ampicillin	Chloramphenicol	This study
pTS127	pTP63-rbs$_{auxB}$-FLAG-auxB^{C25S,C95S,F100C} under a P$_{tet}$ promoter	Ampicillin	Chloramphenicol	This study
pTS163	pLOW-rbs$_{rpoB}$-Myc-PknB$^{\Delta Y376\text{-}V664}$ under a P$_{spac}$ promoter	Ampicillin	Erythromycin	This study
pTS182	pLOW-rbs$_{rpoB}$-Myc-PknB^{K39G} under a P$_{spac}$ promoter	Ampicillin	Erythromycin	This study
pTS203	pLOW-rbs$_{rpoB}$-Myc-PknB$^{K39G,\Delta Y376\text{-}V664}$ under a P$_{spac}$ promoter	Ampicillin	Erythromycin	This study

**Oligo**	**Sequence (5' to 3')**
AGS-2	AGTCAGGAATTCTTACTTGTCGTCATCGTCTTTGTAGTC
AGS-3	AGTCAGGGTACCCTATAAGGAGTTGTAACGAATGAGTAAAGGAGAAGAACTTTTC
AGS-11	AGTCAGGATCCCTACTTGTCGTCATCGTCTTTGTAGTC
AGS-12	AGTCAGGTCGACCATAATTTTTGAGGGGTGAATCTGTATGAGTAAAGGAGAAGAACTTTTC
AGS-17	AGTCAGGGATCCATAGGTAAAATAATAAATGAACGAT
AGS-18	AGTCAGCCTGCAGGTTATACATCATCATAGCTGACTTC
AGS-19	AGTCAGCATATGGACTACAAAGACGATGACGACAAG
AGS-21	AGTCAGGGTACCTTATTCTTGCTCTTTTTTGTCCTTAACTTC
AGS-114	GTCAGGTCGACCATAATTTTTGAGGGGTGAATCTGTATGGACTACAAAGACGATGACGAC
AGS-115	AGTCAGGAATTCTTATTCTTGCTCTTTTTTGTCCTTAACTTC
AGS-117	TGCGTTAGTCAACGTCGAATGAATTTTG
AGS-119	GCTGTCTTCGGTATCGTCGT
AGS-120	ACGTTGACTAACGCAGCTAGGTTGATTTTTCCTACGTTCT
AGS-121	GATACCGAAGACAGCTCATGT
oTS091	ATGGACTACAAAGACGATGACGACAAGACTGGAGAACAATTTACTCAAATTAA
oTS092	TGATGGTACCTAAGTACTATAAGGAGTTGTAACGAATGGACTACAAAGACGATGAC
oTS093	CGGCGCTNAGCTTATTCTTGCTCTTTTTTGTCCTTA

oTS094	CGGCGCTNAGCTTAGTAAATTTGACGTCGCTCTTC
oTS109	TTGTTGATTTCATTTTTAGCTCTCA
oTS113	AGATAACCAACCTAGAACTTTTTCAGT
oTS114	AAGTTCTAGGTTGGTTATCTTGGGTGATGTTATTAGTGCTTAC
oTS115	GCTAAAAATGAAATCAACAAAGAAACAATAAAGTAAACGATAATAGCCC
oTS125	TACTGGACGTTTAATTTGAGTAAAT
oTS127	TGTGTTATTACTGAATGAAACAAGT
oTS128	TTTTTGAGGGGTGAATCTGTATGGACTACAAAGACGATGAC
oTS129	ACAGATTCACCCCTCAAAAAT
oTS134	CAGTGAATTCTTATTCTTGCTCTTTTTTGTCCTTA
oTS135	TGTAGATTAACTGAAAAAGTTCTAGGTT
oTS136	TGTATTGCTAATCTTGAAAATACATTAAACA
oTS137	GCAAGAATAAGAATTCACTGGCCGTCG
oTS163	AAAGCTAAGAAAAAAGAAAAACGTAA
oTS164	TTGTGGCGCAACCATTT
oTS165	ACTGTATATGATCAAGAACAGGAA
oTS166	TTGTGGCGCAACCATTT
oTS167	GCTAAATACAAACAAAAAAGTACTGA
oTS168	ATTTGATTTATCCAATTCTTTTTCATATTTAG
oTS207	GTATTATCCGCTCAGGCTAAATACAAACAAAAAAGT
oTS208	TTAGCCTGAGCGGATAATACTGATGGTTCTTCCT
oTS219	AATGGTATAGCTGCTATTATCGTTTACTTTATTGTTTGT
oTS220	ATAATAGCAGCTATACCATTTTGTAACCATATTACAAATTG
oTS249	CTCGCGGCTATTGAAGGTGATAAAGTATGACAATGACCTAAGAGGTGTGG
oTS250	GAAACCTCGGTACATTTACTTCAATTATATCATAAAAACAACTCGTAGCTTATCAAAG
oTS254	GCTGAAAAAGAAGTCAGCTATGATGATGTAGACTACAAAGACGATGACGACAAGTAACAATGACCT AAGAGGTGTGG
oTS295	AGAAGCTGATCTCTGAAGAGGACCTGATAGGTAAAATAATAAATGAACGATATAAAATTG
oTS296	CGACGGCCAGTGAATTCTTATACATCATCATAGCTGACTTCTT
oTS297	TAAGAATTCACTGGCCGTCG
oTS298	CTCTTCAGAGATCAGCTTCTGTTCCATACAGATTCACCCCTCAAAAAT
oTS359	CGTTTACTTTTGTGTTTCTTTGTTGATTTCATTTTTAG
oTS360	AAGAAACACAAAAGTAAACGATAATAGCCC
oTS361	GTTTCTTTGTGTATTTCATTTTTAGCTCTCATATCTATG
oTS362	AATGAAATACACAAAGAAACAATAAAGTAAACGAT
oTS363	TTGATTTCATGTTTAGCTCTCATATCTATGAATATAAG
oTS364	GAGAGCTAAACATGAAATCAACAAAGAAACAATAA

oTS373	AATGGTATATGTGCTATTATCGTTTACTTTATTGTTTCT
oTS374	ATAATAGCACATATACCATTTTGTAACCATATTACAAATTG
oTS435	AATACCGAGACTTCCAAATAGAAACCTCGGTACATTTACTTCAATTATATCATAAAAACAACTCGT AGCTTATCAAAG
oTS436	CAGCAAGTTATATTGTTAAAGTTGACGGTAAAACTGTAGCTGAAAAAGAAGTCAGCTATG
oTS437	CCGCAATCTCAACATAAAAATACCGAGACTTCCAAATAGAAACCTCGGTACATTTACTTC
oTS608	CGACGGCCAGTGAATTCTTATTTATTACCAAACATTGCCATTG
oTS631	GTTGCAATTGGTGCGATTTTTATACCACCTAGAGAAAAAG
oTS632	AAAATCGCACCAATTGCAACTTTAATGTTAAGTATCGTATCT
AMo81	CCACTGGTAACAGGATTAGCA
AMo82	AATCCTGTTACCAGTGGCT
AMo83	AGACAGCTCATGTTATATCCCG
AMo84	TATAACATGAGCTGTCTTCGGTAT
oWB48	GACGCCGTAAATGGCCTATTGGATGTAGGAGGAGCGCAGTGACCTTCGTGTCCGTGATGGTAAC TTCACG
oWB49	TAACGTATCAGTTGCCGGGCCAGCTACCTTGACGGTTTCCTTGTTGGCTAAGCCGTCAATTGTCT GATTC

References

1. Filsinger GT, Mychack A, Lyerly E, Henriksen C, Bartlett TM, Kuchwara H, Eitzinger S, Bernhardt TG, Walker S, Church GM. 2025. A diverse single-stranded DNA–annealing protein library enables efficient genome editing across bacterial phyla. Proceedings of the National Academy of Sciences 122:e2414342122.
2. Pang T, Wang X, Lim HC, Bernhardt TG, Rudner DZ. 2017. The nucleoid occlusion factor Noc controls DNA replication initiation in Staphylococcus aureus. PLOS Genetics 13:e1006908.
3. Hart EM, Lyerly E, Bernhardt TG. 2024. The conserved σD envelope stress response monitors multiple aspects of envelope integrity in corynebacteria. PLoS Genetics 20:e1011127.
4. Do T, Schaefer K, Santiago AG, Coe KA, Fernandes PB, Kahne D, Pinho MG, Walker S. 2020. Staphylococcus aureus cell growth and division are regulated by an amidase that trims peptides from uncrosslinked peptidoglycan. Nature Microbiology 5:291-303.
5. Coe KA, Lee W, Stone MC, Komazin-Meredith G, Meredith TC, Grad YH, Walker S. 2019. Multi-strain Tn-Seq reveals common daptomycin resistance determinants in Staphylococcus aureus. PLoS pathogens 15:e1007862.
6. Baumgart M, Unthan S, Rückert C, Sivalingam J, Grünberger A, Kalinowski J, Bott M, Noack S, Frunzke J. 2013. Construction of a prophage-free variant of Corynebacterium glutamicum ATCC 13032 for use as a platform strain for basic research and industrial biotechnology. Applied and environmental microbiology 79:6006-6015.
7. Koo B-M, Kritikos G, Farelli JD, Todor H, Tong K, Kimsey H, Wapinski I, Galardini M, Cabal A, Peters JM, Hachmann A-B, Rudner DZ, Allen KN, Typas A, Gross CA. 2017. Construction and Analysis of Two Genome-Scale Deletion Libraries for Bacillus subtilis. Cell Systems 4:291-305.e7.
